# Facing the phase problem

**DOI:** 10.1107/S2052252523006449

**Published:** 2023-09-01

**Authors:** Wayne A. Hendrickson

**Affiliations:** a Department of Biochemistry and Molecular Biophysics, Columbia University, New York, NY 10032, USA; University of Auckland, New Zealand

**Keywords:** anomalous diffraction, density modification, direct methods, isomorphous replacement, molecular replacement

## Abstract

The marvel of X-ray crystallography is the beauty and precision of the atomic structures deduced from diffraction patterns. Since these patterns record only amplitudes, phases for the diffracted waves must also be evaluated for systematic structure determination. Thus, we have the phase problem as a central complication, both intellectually for the field and practically so for many analyses. Here, I discuss how we – myself, my laboratory and the diffraction community – have faced the phase problem, considering the evolution of methods for phase evaluation as structural biology developed to the present day. During the explosive growth of macromolecular crystallography, practice in diffraction analysis evolved from a universal reliance on isomorphous replacement to the eventual domination of anomalous diffraction for *de novo* structure determination. As the Protein Data Bank (PDB) grew and familial relationships among proteins became clear, molecular replacement overtook all other phasing methods; however, experimental phasing remained essential for molecules without obvious precedents, with multi- and single-wavelength anomalous diffraction (MAD and SAD) predominating. While the mathematics-based direct methods had proved to be inadequate for typical macromolecules, they returned to crack substantial selenium substructures in SAD analyses of selenomethionyl proteins. Native SAD, exploiting the intrinsic S and P atoms of biomolecules, has become routine. Selenomethionyl SAD and MAD were the mainstays of structural genomics efforts to populate the PDB with novel proteins. A recent dividend has been paid in the success of PDB-trained artificial intelligence approaches for protein structure prediction. Currently, molecular replacement with *AlphaFold* models often obviates the need for experimental phase evaluation. For multiple reasons, we are now unfazed by the phase problem. Cryo-EM analysis is an attractive alternative to crystallography for many applications faced by today’s structural biologists. It simply finesses the phase problem; however, the principles and procedures of diffraction analysis remain pertinent and are adopted in single-particle cryo-EM studies of biomolecules.

## Abstract 2

This article reviews phase evaluation in macromolecular crystallography. It accompanies the 2023 Ewald Prize lecture at the 26th IUCr Congress, paying tribute to Paul Ewald’s far-reaching influence.

## 1. Introduction

The momentous discovery of X-ray diffraction by Friedrich, Knipping and Laue (Friedrich *et al.*, 1912 ▸) has had manifold ramifications. Atomic structure determination is prominent among these, and is arguably pre-eminent. In favorable circumstances, which are not at all atypical, the number of unique reflections in the diffraction from a crystal exceeds the number of parameters needed to define its atomic structure, often manyfold overdeterminatively. Diffraction amplitudes can be measured accurately, whereby X-ray crystallography becomes a manifestly definitive science. However, since the atomic parameters relate nonlinearly to diffraction patterns, structure solution requires having an initial model within the radius of convergence of a minimization procedure that can relate the atomic model to the diffraction data. For complicated structures, such as those of biological macromolecules, suitable initial models are built to fit electron-density distributions from Fourier syntheses, which require phase angles as well as the diffraction amplitudes. Thus, we face the phase problem.

## 2. Discovery of X-ray diffraction and structure determination

The announcement of Laue’s discovery that crystals can diffract X-rays was followed quickly by contributions from Bragg (1913*a* ▸) and Ewald (1913 ▸) that clarified the conditions for diffraction: (**S** · **a** = *h*; **S** · **b** = *k*; **S** · **c** = *l*) by Laue, where **S** = **s** – **s**_0_ is the diffraction vector, **a**, **b** and **c** are unit-cell vectors and *h*, *k* and *l* are integers; *n*λ = 2*d*sinθ by Bragg, where λ is the wavelength, *d* is the spacing between ‘reflecting’ planes, θ is the reflection angle and *n* is an integer; and by Ewald as when the reciprocal-lattice vector **S** = *h* a* + *k* b* + *l* c* intercepts the **s**_0_-directed sphere of radius 1/λ, where a*, b* and c* define the reciprocal unit cell and |**S**| = 2sinθ/λ = 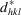 = 1/*d*_*hkl*_.

A series of papers followed shortly from the Braggs deducing the atomic structures of salts and minerals from their diffraction patterns (Bragg, 1913*b* ▸, 1914 ▸; Bragg & Bragg, 1913 ▸). These initial structures, including NaCl and diamond, were of face-centered cubic crystals with all atomic positions specified by the lattice symmetry. Pyrite (FeS_2_) and calcite (CaCO_3_) each have one free parameter, which Bragg determined by comparing relative intensities computed from alternative models with measured intensities. This approach, which came to be known as ‘trial and error’, was then used in many-parameter structural analyses including those for diopside [CaMg(SiO_3_)_2_] (Warren & Bragg, 1929 ▸) and benzene derivatives (Lonsdale, 1929 ▸, 1931 ▸). These early structures were determined by the logic of symmetry and by constraints from especially intense reflections (notably Lonsdale, 1929 ▸), but the phases associated with the reflected X-ray waves were not invoked.

## 3. Fourier transformation and the phase problem

Images form coherently as scattered waves are collected and then recombined by the optical lens of light microscopes or the electromagnetic lenses of electron microscopes. This is not directly possible for the X-ray diffraction experiment, however, since there are no lenses for hard X-rays; the phases of X-ray waves are lost as the X-ray diffraction is measured. Abbe (1873 ▸) described and tested a diffraction theory of image formation in the light microscope, and Porter (1906 ▸) provided a mathematical foundation for this double-Fourier-transform theory, which Glaeser *et al.* (2007 ▸) elaborated for the electron microscope. W. H. Bragg picked up on the ideas of Porter (Bragg, 1915 ▸) and suggested Fourier analysis for X-ray diffraction, but it was Ewald (1921 ▸) who formulated Fourier transformation in practical terms of the reciprocal lattice. Duane (1925 ▸) rediscovered electron-density reconstruction by Fourier synthesis. While not directly experimental, this computational approach to the completion of image formation does have the advantages of being free from lens distortions and of offering complete three-dimensional reconstructions.

Bragg (1929 ▸) implemented the Ewald (1921 ▸) formulation of inverse Fourier transformation to produce images for diopside, using the known structure (Warren & Bragg, 1929 ▸) to compute phases (actually signs since this structure is centrosymmetric). These appealing images were highly influential, taking hold immediately in structure completion (Lonsdale, 1931 ▸), especially so with the innovation of the efficient Fourier strips of Beevers & Lipson (1934 ▸). Moreover, systematically direct structure determination would now be possible provided that phases could be determined. Comparison of diffraction from isomorphous series provided the basis for determining phases in the pioneering Fourier analyses of alums (Beevers & Lipson, 1935 ▸) and phthalocyanins (Robertson, 1936 ▸). When the ‘phase problem’ was first posed as such is unclear; however, as Patterson (1934 ▸) introduced his |*F*|^2^ series, he plainly identified the determination of phases as **the** problem of X-ray crystal analysis. Certainly, it stood pre-eminent by the time that Hauptman and Karle boldly titled their ACA Monograph *Solution of the Phase Problem I. The Centrosymmetric Crystal* (Hauptman & Karle, 1953 ▸).

## 4. Phase evaluation in the small-molecule tradition

With the adoption of Fourier methods, crystal structure determinations advanced from the simple to the complex, from projections to three-dimensional syntheses, and from centrosymmetric to noncentrosymmetric structures. For a couple of decades, these applications remained in the realm of what we now call small molecules; however, methods from this small-molecule tradition greatly influenced the approaches taken in macromolecular crystallography.

In many cases, the scattering dominance of higher *Z* elements can be the basis for phase evaluation, either by the isomorphous replacement method or by the heavy-atom method. The Fourier analysis of phthalocyanin from comparison of the apo and nickel-substituted forms (Robertson, 1936 ▸) typifies the former approach, and the analysis of platinum phthalocyanin (Robertson & Woodward, 1940 ▸) exemplifies the latter. These structures are centrosymmetric and simplified by having the metal ion at the origin. The structure of cholesteryl iodide is noncentrosymmetric, but the general position of its I atom was readily evident in the Patterson synthesis of a centrosymmetric projection, and the iodine-phased Fourier synthesis revealed the sterol structure (Carlisle & Crowfoot, 1945 ▸). Structures of the alkaloid strychnine were determined in one case using bromine as a heavy atom in combination with Patterson methods (Robertson & Beevers, 1951 ▸) and in another by selenium versus sulfur isomorphous replacement (Bokhoven *et al.*, 1951 ▸). The latter entailed the complication of unspecified phase alternatives, which prompted the suggestion of double substitutions (selenium for sulfur and bromine for chlorine) to resolve the phase ambiguity (Bokhoven *et al.*, 1951 ▸).

Higher *Z* elements, intrinsically present but not dominating the diffraction, sufficed (along with perseverance and chemical intuition) to solve the challenging structures of penicillin (Crowfoot *et al.*, 1949 ▸) and vitamin B_12_ (Hodgkin *et al.*, 1957 ▸). However, most organic molecules only have atoms of quasi-equal scattering strength (C, N and O, with H being a feeble scatterer), and the addition of heavy atoms (as was performed for cholesterol and strychnine) is not always readily feasible. Fortunately, the need for equal-atom phasing methods was met by the development of direct methods. The number of unique diffraction observations from a typical small-molecule crystal greatly exceeds the number of independent atomic parameters; thus, the reflections must be interrelated. Indeed, both the positivity-based determinants of Karle & Hauptman (1950 ▸) and the atomicity-based Sayre’s equation (Sayre, 1952 ▸) demonstrate interactions among triples of structure factors *F*(**h**), *F*(**k**) and *F*(**h** − **k**), where **h** (*h*, *k*, *l*) is a reciprocal-lattice vector. Moreover, from order-three determinants (Karle & Hauptman, 1950 ▸), the phase relationship φ(**h**) ≃ φ(**k**) + φ(**h** − **k**) holds for triplets with amplitudes [|*F*(**h**)|, |*F*(**k**)| and |*F*(**h** − **k**)|] of sufficient strength. The probabilities for these and other such relationships (Hauptman & Karle, 1953 ▸; Karle & Hauptman, 1956 ▸) founded the basis for routinely effective phasing procedures for quite substantial equal-atom structures.

## 5. Resonance and anomalous scattering

Anomalous diffraction had little impact in small-molecule crystallography, but its potential for phase determination did become appreciated and tested during this pre-macromolecule era (Bijvoet, 1949 ▸, 1954 ▸; Okaya & Pepinsky, 1956 ▸; Ramachandran & Raman, 1956 ▸). Some time earlier, Kramers (1924 ▸) had already anticipated anomalous dispersion due to resonance with electronic transitions in the atomic model of Bohr (1913 ▸), and Mark & Szilard (1925 ▸) had characterized such effects in the diffraction from rubidium bromide.

The lattice of RbBr is face-centered cubic, as for NaCl and other alkali halides; however, since Rb^+^ and Br^−^ are iso­electronic, ‘normal’ diffraction from RbBr is as if from an *a*/2 primitive cube. Only even orders of [111] reflections are present for ‘normal’ X-ray scattering, as from Cu *K*α radiation. With Sr *K*α X-rays, chosen for having an energy (14.1 keV) between the rubidium (13.5 keV) and bromine (15.21 keV) absorption edges, the two sites then scatter distinctively and reflections from (111) and (333) are observed. On considering the phase shifts for such phenomena (Ewald & Hermann, 1927 ▸), Ewald retracted his earlier ‘proof’ (based on the theory for dynamical diffraction, but not yet contemplating anomalous scattering) that Friedel’s law always holds. Somewhat later, Coster, Knol and Prins famously bracketed the zinc absorption edge with gold lines to show pronounced differences between the (111) and (1 1 1) reflections from zincblende (ZnS) for the ratios of intensities from Au *L*α1 X-rays above the Zn *K* edge versus from Au *L*α2 X-rays below the edge (Coster *et al.*, 1930 ▸).

**Figure 1:**
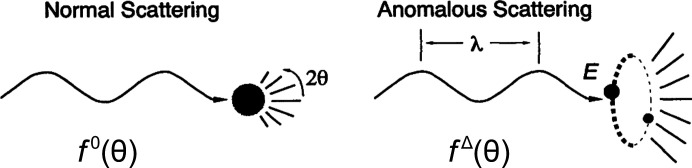
The atomic scattering factor describes the coherent scattering of X-rays from a given atom. This has two additive components (equation 1): the normal component, *f* ^0^, from Thomson scattering decreases smoothly with the Bragg angle θ but has no intrinsic dependence on the wavelength, and the anomalous component, *f* ^Δ^, from resonance with electron transitions has a sharp dependence on the wavelength λ near absorptive transitions but is nearly constant with respect to the scattering angle. Adapted from Box 1 of Hendrickson (1991 ▸) with permission from AAAS.

‘Anomalous’ scattering arises from resonance between the X-ray energy and the transition energy between a ground-state core orbital and an unoccupied outer orbital, which necessarily is a quantum phenomenon (Hönl, 1933 ▸; Als-Nielsen & McMorrow, 2011 ▸). The classical theory of Thomson (1906 ▸) for elastic scattering of X-rays from a free electron also applies to X-ray scattering from electrons bound into atomic orbitals but, of course, the electron-density distributions of atoms are also quantal, ρ = ψψ*. The two processes (Fig. 1 ▸) are additive. Altogether, the atomic scattering factor, *f*, comprises a ‘normal’ component due to the Thomson scattering, *f* ^0^, plus the ‘anomalous’ component, *f* ^Δ^ = |*f* ^Δ^|exp(*i*δ), which entails an incremental phase shift, δ, beyond the shift of π/2 that accompanies Thomson scattering. In principle, resonant scattering is the actual norm; in practice, however, the X-ray energy is often remote from absorption edges, and anomalies such as departure from Friedel’s law are slight.

Because core electronic orbitals are confined near to the nucleus, whereas outer-shell electrons are relatively diffuse, the normal scattering decreases smoothly with diffraction-vector amplitude *S* = |**S**| = 2sinθ/λ. Thereby, *f* ^0^ depends on scattering angle but not directly on wavelength. By contrast, *f* ^Δ^ barely changes with scattering angle but depends sharply on wavelength when near the absorption-edge resonance. Thus,

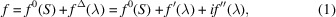

where *f*′(λ) and *f*′′(λ) are the real and imaginary components of the anomalous scattering, respectively. The imaginary component of forward scattering extinguishes part of the incident beam, and is thereby absorptive; thus, *f*′′(*E*) = *K*μ(*E*)*E*, where μ is the absorption coefficient. Electrons ejected by photoelectric absorption are recaptured proportionately with absorption, and fluorescent photons are emitted as this happens. This provides a means for measuring *f*′′ spectra. In turn, the *f*′ spectrum is determined from the *f*′′ values by Kramers–Kronig transformation (Kronig & Kramers, 1928 ▸).

## 6. Evaluation of phases in macromolecular crystallography

Contemporaneous with the afore­mentioned advances in structure determination and demonstrations of anomalous scattering, diffraction experiments were also beginning for proteins. It was obvious from diffraction patterns of properly hydrated crystalline pepsin (Bernal & Crowfoot, 1934 ▸) that structures of ‘a perfectly definite kind’ could come from such patterns, but that such structures would not comprise the periodicities found by Astbury for α-type and β-type fibrous proteins (Astbury & Street, 1931 ▸). From the large unit-cell dimensions, it was doubtless clear as well that projections would be of little use and that general phases (not signs) would be required. It would take some while for protein crystal structures to be solved. Meanwhile, diffraction-constrained model building provided structural models for the constituent α-helices (Pauling *et al.*, 1951 ▸) and β-sheets (Pauling & Corey, 1951 ▸) of proteins. These models took the molecular geometry from Pauling’s crystal structures of amino acids and dipeptides, and their approach also paved the way for the double-helix DNA structure built by Watson & Crick (1953 ▸) to explain Franklin’s diffraction patterns from B-form DNA (Franklin & Gosling, 1953 ▸).

**Figure 2:**
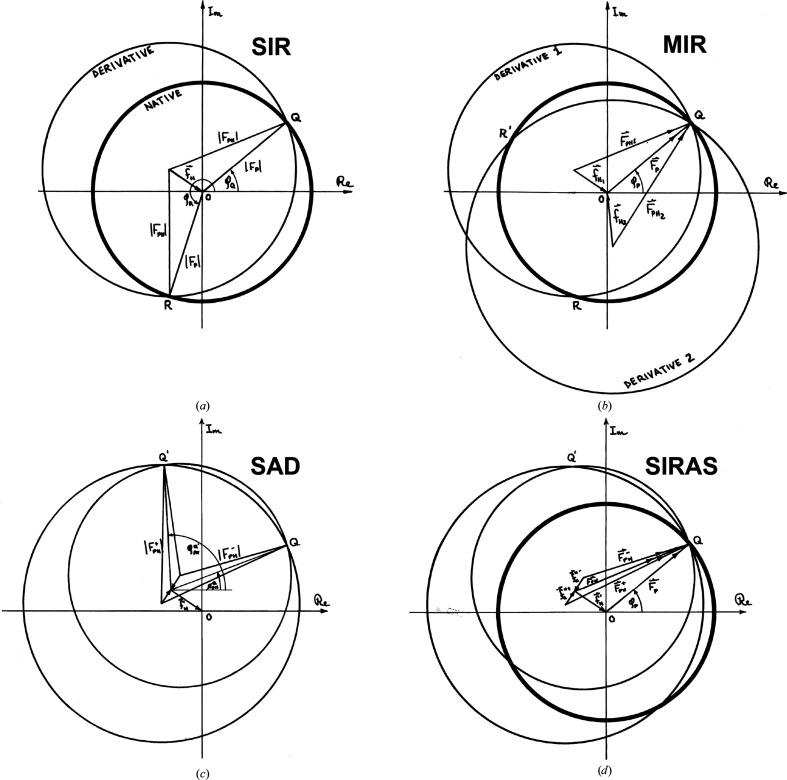
Harker phasing diagrams illustrating the phase ambiguity inherent in SIR phasing (*a*) and SAD phasing (*c*), and its resolution by two derivatives in MIR (*b*) or by SIRAS (*d*). The ‘native’ amplitude circles (heavy lines) each have radius |*F*_P_|. The single isomorphous pair from SIR derivative 1 yields options at *Q* or *R*, whereas derivative 2 gives alternatives *Q* and *R*′. The anomalous scattering from derivative 1 gives alternatives *Q* and *Q*′. Neglecting experimental error, in principle either MIR or SIRAS resolves the phase ambiguity. Adapted from Figs. 4 and 6 of Hendrickson (1981 ▸) with permission from Macmillan.

What emerged for evaluating phases for diffraction from protein crystals was the method of multiple isomorphous replacement (MIR). Perutz and coworkers showed that effects from Hg atoms added to hemoglobin crystals could suffice for phase determination (Green *et al.*, 1954 ▸; Blow, 1958 ▸). Although phase information from a single heavy-atom derivative is ambiguous, in general giving two alternatives for each reflection, a second distinctive derivative could resolve the ambiguity, as suggested in Bijvoet’s analysis of strychnine (Bokhoven *et al.*, 1951 ▸) and elaborated algebraically and graphically by Harker (1956 ▸). Harker’s phasing diagram illustrated the ambiguity from a single isomorphous derivative [SIR; Fig. 2 ▸(*a*)] and how this can be resolved by including a second derivative [MIR; Fig. 2 ▸(*b*)]. Blow & Rossmann (1961 ▸) showed that the anomalous scattering from a single heavy-atom derivative [Fig. 2 ▸(*c*)] could be used to resolve the phase ambiguity [SIRAS; Fig. 2 ▸(*c*)], and North (1965 ▸) and Matthews (1966 ▸) elaborated its use in the isomorphous replacement method. MIR with anomalous scattering (MIRAS) was first mentioned for the structure of reduced hemoglobin (Muirhead & Perutz, 1963 ▸), and single isomorphous replacement with anomalous scattering (SIRAS) was first used for the structure of rubredoxin (Herriott *et al.*, 1970 ▸).

Algebraically, the isomorphous-replacement differences with a single derivative are

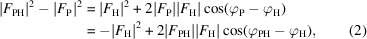

where *F*_P_(**h**) = |*F*_P_|exp(*i*φ_P_), *F*_PH_(**h**) = |*F*_PH_|exp(*i*φ_PH_) and *F*_H_(**h**) = |*F*_H_|exp(*i*φ_H_) are the respective structure factors for reflection **h** (*h*, *k*, *l*) from the parent protein P, the isomorph PH derivatized with heavy atom H, and the heavy-atom contribution H to PH. When anomalous scattering is present from a single kind of scatterer, the anomalous diffraction differences from Friedel mates (or Bijvoet equivalents) are

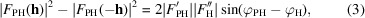

where 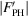 ≃ ½[|*F*_PH_(**h**)| + |*F*_PH_(−**h**)|] is the real component of |*F*_PH_| and 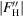 is the imaginary component of |*F*_H_| (Hendrickson, 1979 ▸). The phase ambiguities are clearly evident from the trigonometric factors of equations (2) and (3),

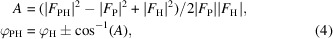

and

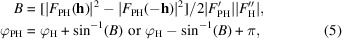

whereas the orthogonality of isomorphous replacement and anomalous diffraction in SIRAS resolves the ambiguity,

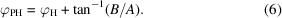




Macromolecular crystallography also borrowed from the small-molecule tradition in other ways besides its adoption of isomorphous replacement. Although the Patterson function was ill-suited to the heavily overlapped interatomic vectors of proteins themselves, it was well suited to the critical problem of finding the positions of heavy atoms in the derivatives. For this, two developments were instrumental: one is the Patterson synthesis with coefficients ||*F*_PH_| − |*F*_P_||^2^ (Rossmann, 1960 ▸) or ||*F*_PH_(**h**)| − |*F*_PH_(−**h**)||^2^ (Rossmann, 1961 ▸), which approximate to the Patterson maps for |*F*_H_|^2^, and the other concerns special symmetry-related Patterson sections (Harker, 1936 ▸), *v* = ½ for space group *P*2_1_ for example, whereby certain atomic co­ordinates can be read off directly from self-vectors between symmetry mates. Similarly, while macromolecules themselves are out of the reach of direct methods, the substructures of heavy atoms are accessible from normalized difference co­efficients, as shown by Steitz (1968 ▸) and now used routinely (Schneider & Sheldrick, 2002 ▸).

**Figure 3:**
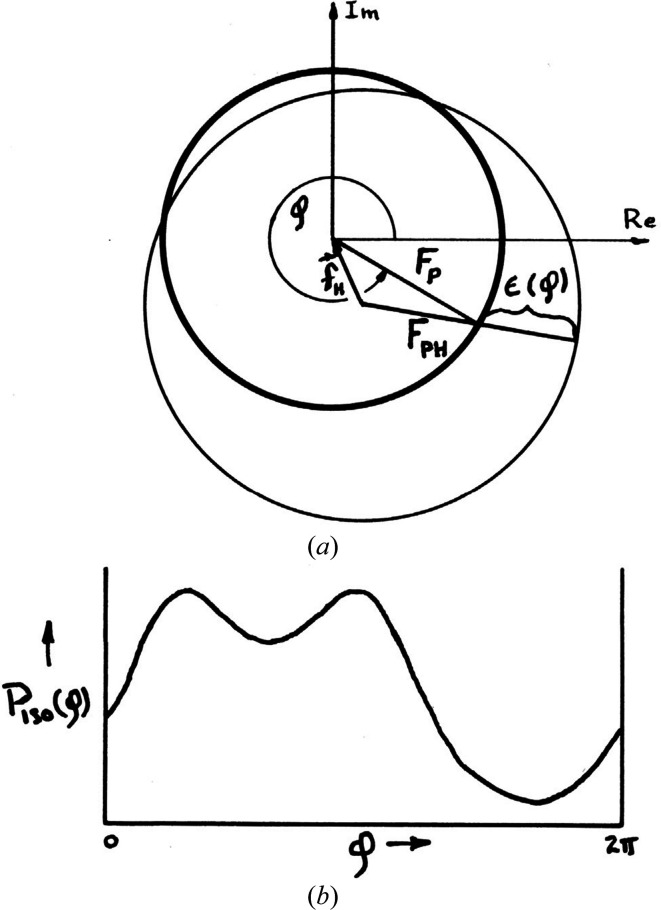
The phase probability analysis of Blow & Crick (1959 ▸) by lack-of-closure error ɛ(φ), equations (7) and (8). (*a*) Harker diagram illustrating the lack-of-closure error. (*b*) Phase probability associated with the single isomorphous derivative in (*a*). Reproduced from Fig. 5 of Hendrickson (1981 ▸) with permission from Macmillan.

Harker diagrams (Harker, 1956 ▸) were used in manual phase evaluations for the structure of myoglobin at 6 Å resolution (Kendrew *et al.*, 1958 ▸), which employed five heavy-atom derivatives. This approach proved laborious and fraught (Bodo *et al.*, 1959 ▸), and Blow & Crick (1959 ▸) supplanted it by a probability treatment that introduced the lack-of-closure error (Fig. 3 ▸) and the ‘best Fourier’ as the probability-weighted inclusion of all phase angles. This Blow–Crick lack-of-closure error is defined as

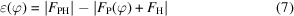

and the phase probability distribution is

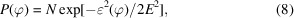

where the standard error *E* ^2^ is the variance for lack of closure for optimal (centroid) phase angles, φ_c_, which in practice is arrived at by iteration, *E* ^2^ = 〈ɛ^2^(φ_c_)〉.

Assuming the independence of information from individual derivatives, the combined probability for multiple isomorphous replacements becomes the product of the individual probabilities. We found that the Blow–Crick formulation (equations 7 and 8) defied analytic reduction to a simplified representation for encoding phase information for facile storage and combination (Hendrickson & Lattman, 1970 ▸), but that a related alternative closure definition would do so:

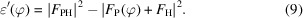

The Gaussian form of (8) then gives the phase probability in terms of ɛ′ and its associated variance, 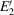 , which was shown by Blundell & Johnson (1976 ▸) to be related to the Blow–Crick variance as

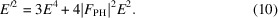

With this alternative definition for closure discrepancy, the phase information from isomorphous replacement can define the phasing coefficients *A*, *B*, *C* and *D* for the distribution

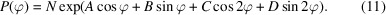

The formalism of (11), from which the phasing integrals can be evaluated analytically, also straightforwardly accommodated phase information from other sources, namely anomalous scattering, direct methods, noncrystallographic symmetry and partial structures.

Blundell & Johnson (1976 ▸) superbly chronicled the methods by which large numbers of macromolecular structures had already been determined; however, at that time many mainstays of biological crystallography were yet to come. The early analyses relied almost exclusively on isomorphous replacements for phase evaluation, albeit often supplemented by information from anomalous scattering. Effective structure-refinement methods had not yet taken hold (Jack & Levitt, 1978 ▸; Hendrickson & Konnert, 1980 ▸; Konnert & Hendrickson, 1980 ▸; Sussman, 1985 ▸; Brünger, 1991 ▸). Methods for cDNA cloning and manipulation were just being invented, but recombinant protein expression was not available. Synchrotron radiation was known (Rosenbaum *et al.*, 1971 ▸), but crystallography beamlines did not exist. The rotation function was known (Rossmann & Blow, 1962 ▸) and molecular-replacement trials had been made (Tollin, 1969 ▸; Lattman *et al.*, 1971 ▸), but the first atomic-level extension was then only in press (Schmid *et al.*, 1974 ▸; Schmid & Herriott, 1976 ▸). Phasing from noncrystallographic symmetry had been anticipated (Rossmann & Blow, 1963 ▸), but density modification by molecular averaging, as symmetry is now exploited, and by solvent flattening were for the future. Direct methods had been tried for phase refinement (Hendrickson & Karle, 1973 ▸; Sayre, 1974 ▸), but the improvements were not striking. Without the continuous X-ray spectra from synchrotrons, truly effective use of resonance was not yet possible.

## 7. Phase information from partial structures

Although heavy atoms were highly effective in isomorphous replacement phasing, they did not suffice for direct heavy-atom phasing of macromolecules. Later, as the body of known structures grew and the molecular-replacement method developed, phase information from partial structures became increasingly important at intermediate stages of structure determination. The Sim probability formula (Sim, 1960 ▸) implicitly assumes an error-free partial structure, which effectively is the case for heavy atoms; however, partial structures from molecular replacement often deviate appreciably from the correct answer. A reliable formulation for structure completion needs to take this into account, as was anticipated by Rossmann & Blow (1961 ▸) and noted by us (Hendrickson & Lattman, 1970 ▸); however, it was not until Read (1986 ▸) implemented effective σ_A_ estimates for the formulation of Srinivasan (1966 ▸) that partial structure development was placed on a firm footing.

Following the nomenclature of Sim (1960 ▸), the total structure of *N* atoms is divided into the *P* atoms of the partial structure model and *Q* missing atoms. The expected contribution from the missing atoms is 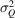 = 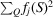 = 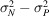 . To also account for the missing contribution due to errors in the partial model, Srinivasan (1966 ▸) introduced an error factor due to Luzzati (1952 ▸) into a Sim-like formulation, following developments by Vand & Pepinsky (1957 ▸) and Srinivasan & Ramachandran (1965 ▸). The phase probability for a given reflection is then

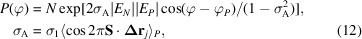

where *N* normalizes for unit integrated probability, |*E*_*N*_| = 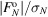 is the normalized structure amplitude observed from all *N* atoms, |*E*_*P*_| = 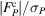 is the normalized structure amplitude calculated from the *P* atoms of the model, **Δr**_*j*_ is the positional error for the *j*th atom of the model and σ_1_ = σ_*P*_/σ_*N*_. With appropriate evaluation of σ_A_ (Read, 1986 ▸), the σ_A_-based treatment of model errors appreciably facilitates the elaboration of partial structures derived both from molecular replacement and from experimental phasing, and coefficients for the simplified representation (Hendrickson & Lattman, 1970 ▸) derive straightforwardly.

## 8. Molecular replacement

Albeit without full elaboration, Rossmann & Blow (1962 ▸) anticipated three uses for the rotation function: (i) the characterization of molecular symmetry, (ii) phase determination from noncrystallographic symmetry and (iii) the repositioning of a molecule known from one crystal lattice into another lattice. They immediately used the rotation function to characterize the approximate symmetry relating the α and β subunits of hemoglobin, and, in this vein, we used it to exhibit pseudosymmetry within myohemerythrin (Hendrickson & Ward, 1977 ▸). Elegant formulations for the phase information from noncrystallographic symmetry followed (Main & Rossmann, 1966 ▸; Crowther, 1967 ▸); however, real-space averaging proved simpler to effect, and such phase refinement has become a staple of density modification. Rossmann (1990 ▸) persisted in using ‘molecular replacement’ to describe phasing from noncrystallographic symmetry, but prevailing crystallo­graphic parlance restricts the term to the third category of usage. Demand for such applications awaited the accumulation of known structures and appreciation of the value in analyses of evolutionary homologs and nonisomorphic variants of these knowns. This use gave us a phasing method unique to macromolecules, going beyond the small-molecule tradition, and it has come to prevail in macromolecular crystallography.

Molecular replacement, in this usual sense of the term, borrows the phases from a molecule of known structure to determine the crystal structure of an unknown candidate that includes a component structurally similar to this known. An orthogonal transformation is sought to reposition the known structure, {**r**_*A*_}, into the lattice of the unknown: {**r**_*B*_} ≃ {**r**_*A*′_} = **R**{**r**_*A*_} + **t**, where **R** and **t** are the rotation matrix and translation vector, respectively. The new structure is then composed by Fourier synthesis with phases from the previously known structure, as so transformed: ρ_*B*_(**r**) ≃ 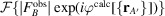 . Rigid-body refinement improves the transformation, and partial structure methods combined with density modification (described below for SAD ambiguity resolution) can remove bias toward the known calculation. We used molecular averaging and solvent constancy to refine the density for octameric hemerythrin (Ward *et al.*, 1975 ▸) after replacement from the structure of myohemerythrin (Hendrickson *et al.*, 1975 ▸).

The rotation function (Rossmann & Blow, 1962 ▸), typically as fast Fourier transform (FFT) enhanced (Crowther, 1972 ▸), is proficient for finding the rotational orientation. The specification of translation can be more problematic. Early translation functions based on symmetry mates (Crowther & Blow, 1967 ▸) lacked generality and often proved inconclusive, packing functions have rather low resolution (Hendrickson & Ward, 1976 ▸), and brute-force variation is inefficient. Nevertheless, various program systems supported the promulgation of the method. Currently, the *Phaser* system (McCoy *et al.*, 2007 ▸), based on maximum-likelihood procedures (Read, 2001 ▸), is commonly used.

## 9. Crambin and the power of anomalous diffraction

The early success of isomorphous replacement in macromolecular crystallography was perpetuated by emulation. The method is robust, tolerating imperfect isomorphism and errors from differencing between diffraction patterns measured from two different crystals. Nevertheless, one would ideally obtain the structure from a single native crystal. Moreover, we were unable to derivatize the well diffracting crystals of crambin that Martha Teeter had discovered (Teeter & Hendrickson, 1979 ▸). The sequence of crambin does include six disulfide-bridged cysteine residues (no methionines), and our experience in using anomalous scattering to locate the Fe atoms in myohemerythrin (Hendrickson *et al.*, 1975 ▸) led us to contemplate a similar approach here as well, despite the expectation of much weaker signals. After estimating the precision that would be required, we then established measurement parameters to assure adequate counting statistics for data collection by single-counter diffractometry with a Cu *K*α X-ray source, for which sulfur has *f*′′ = 0.557 electrons (Cromer & Liberman, 1970 ▸). The diffraction was measured to a Bragg spacing limit of 0.945 Å.

**Figure 4:**
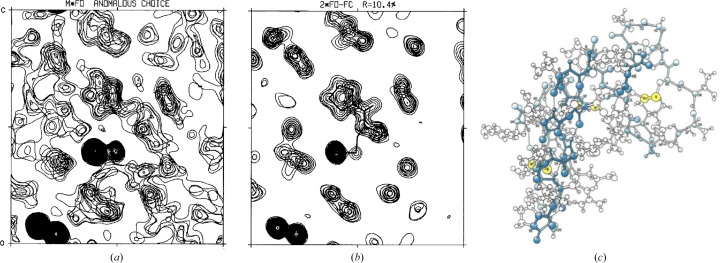
SAD phasing structural analysis of crambin. (*a*) A portion of what would now be called the sulfur SAD map for crambin at 1.5 Å resolution. The densest features are at S atoms. (*b*) The same portion as in (*a*) for the final 2*F*_o_ − *F*_c_ map after refinement to *R* = 0.104 at 1.5 Å resolution. (*c*) All-atom atomic model of crambin drawn by Irving Geis. S atoms are colored yellow and the first helix is filled in blue. (*a*) and (*b*) are adapted from Fig. 2 of Hendrickson & Teeter (1981 ▸) with permission from Springer Nature, and (*c*) is adapted from a drawing by Irving Geis with permission from the Howard Hughes Medical Institute, which now owns the Geis collection.

The positions of S atoms were readily deduced from Patterson maps, first at 3 Å resolution, where disulfide bridges are not resolved, and then at 1.5 Å resolution to separate the six individual S atoms. We computed phase probabilities from a rearranged version of equation (3) and attempted the resolution of phase ambiguities from the S atoms as a partial structure. Partial structure probabilities were computed from Sim’s formula [*i.e.* equation (12) with σ_A_ = σ_1_ and *F*s instead of *E*s] and combined by a probabilistic choice procedure (Hendrickson, 1971 ▸; Hendrickson & Teeter, 1981 ▸). Ambiguity resolution was not fully successful [Fig. 4 ▸(*a*)]; nevertheless, sufficient features were evident to develop a refined structure [Fig. 4 ▸(*b*)] for a complete atomic model [Fig. 4 ▸(*c*)].

Ultimately, we refined crambin anisotropically at the full 0.945 Å resolution of our data (Smith *et al.*, 1986 ▸), and Teeter and coworkers refined the structure against data extended to 0.48 Å resolution (Schmidt *et al.*, 2011 ▸). Sheldrick *et al.* (1993 ▸) showed that the entire structure could be developed without recourse to anomalous scattering from Patterson-derived S atoms, and Hauptman’s group proved that the crambin structure of 418 non-H atoms (including waters) could also be solved directly by their *Shake-n-Bake* (*SnB*) procedure (Weeks *et al.*, 1995 ▸), again without anomalous scattering. Clearly, crambin is an exceptional protein. There also are notable examples of small-molecule phasing based on anomalous scattering (Hall & Maslen, 1965 ▸; Moncrief & Lipscomb, 1966 ▸; Fridrichsons & Mathieson, 1967 ▸; Venkatesan *et al.*, 1971 ▸). All the same, the crambin structure served to demonstrate the exceptional power of anomalous diffraction for phase definition and its potential for directly determining macromolecular structures.

## 10. Multi-wavelength anomalous diffraction (MAD)

In the 1950s ferment of phase-problem attention, various formulations were proposed for phase evaluation from anomalous scattering (Ramachandran & Raman, 1956 ▸; Okaya & Pepinsky, 1956 ▸; Mitchell, 1957 ▸; Raman, 1959 ▸). Tests of ambiguity resolution by analyses at two wavelengths followed (Ramaseshan *et al.*, 1957 ▸; Hoppe & Jakubowski, 1971 ▸); however, in the absence of synchrotron radiation these experiments were limited to the X-ray wavelengths of characteristic emissions. The *Bremsstrahlung* continuum can also generate diffraction, of course, as in the first X-ray diffraction experiments (Friedrich *et al.*, 1912 ▸); indeed, although weak, the *Bremsstrahlung* from a molybdenum anode sufficed to solve the structure of selenolanthionine (Hendrickson, 1985 ▸). In this experiment, we supplemented Mo *K*α with three monochromatic wavelengths selected from the *Bremsstrahlung* to optimize anomalous signals from the Se *K* edge for phase evaluation by our new multi-wavelength anomalous diffraction (MAD) formulation. Templeton & Templeton (1988 ▸) refined the selenolanthionine structure and used synchrotron radiation to characterize the pleochroism of anomalous scattering near the Se *K* absorption edge.

As synchrotrons took hold, fluorescence spectroscopy experiments revealed very sharp features at many absorption edges, from which relevance to diffraction studies was immediately apparent (Phillips *et al.*, 1978 ▸; Templeton *et al.*, 1980 ▸; Lye *et al.*, 1980 ▸). Influenced by our own synchrotron experience in the X-ray absorption spectroscopy of hemerythrin (Hendrickson *et al.*, 1982 ▸) and frustrated by phase ambiguity in the crambin study (Hendrickson & Teeter, 1981 ▸), I then revisited the earlier analyses of anomalous diffraction, aiming for the optimization that synchrotron radiation affords. This led to my formulation of MAD (Hendrickson, 1985 ▸), which we tested in applications to lamprey hemoglobin (Hendrickson, 1985 ▸; Hendrickson *et al.*, 1988 ▸) and which I introduced at the 13th IUCr Congress in Hamburg (Hendrickson, 1984 ▸). We have previously reviewed developments of anomalous diffraction and its many applications (Hendrickson, 1991 ▸, 2014 ▸; Hendrickson & Ogata, 1997 ▸) and I only summarize here.

In principle, all atoms are anomalous scatterers; however, the X-rays used in typical experiments (*E* > 6 keV; λ < 2 Å) are far from resonance for nearly all atoms in a biological macromolecule, whereby their anomalous scattering factors (*f* ^Δ^) are negligible. We ignore the scattering from hydrogen altogether and consider N, C and O atoms to be normal scatterers (N), whereas, for example, the Fe atom in heme is an anomalous scatterer (A). The designation of A versus N is only a matter of relative effect: thus, P and S atoms may be designated N for a metal complex but A in studies of native nucleic acids or metal-free proteins. In our diffraction analysis (Hendrickson, 1985 ▸; Hendrickson *et al.*, 1988 ▸), we separate contributions from the A subset of atoms from the total diffraction (T atoms, both A and N) in a manner of algebraic dissection due to Karle (1967 ▸). We then determine the A substructure and use it to deduce phases for the total diffraction, by which the entire structure should be revealed.

The normal scattering components (*f* ^0^) are wavelength-independent and pure real, whereas the anomalous scattering components (*f* ^Δ^ = *f*′ + *if*′′) are wavelength-variant and complex. Structure factors follow from the contributing scattering-factor components (equation 1); thus, ^λ^ *F*_T_(*f*) = ^0^ *F*_T_(*f* ^0^) + 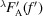 + 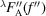 . Superscript prefixes of λ or 0 connote wavelength dependence or independence, respectively. Thus, ^λ^ *F*_T_ is the complete structure factor, which is a function of wavelength, ^0^ *F*_T_ = |^0^ *F*_T_|exp(*i* ^0^φ_T_) = ^0^ *F*_N_ + ^0^ *F*_A_ and ^0^ *F*_A_ = |^0^ *F*_A_|exp(*i* ^0^φ_A_) are the wavelength-invariant normal scattering components contributing to ^λ^ *F*_T_, 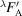 is the structure-factor component from the real parts of anomalous scattering, and 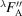 is the structure-factor component from the imaginary parts of anomalous scattering. A macromolecule may contain more than one kind of anomalous scatterer, for example iron in heme and selenium in selenomethionine residues, and contributions of each kind *k* are specified by 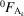 and the scattering factors 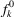 , 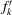 and 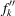 . Thereby, the structure factor for reflection **h** (*h*, *k*, *l*) measured at wavelength λ is given by

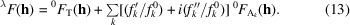




The system of equations (13) for *N* kinds of anomalous scatterers can in principle be solved from diffraction data at 2*N* + 1 wavelengths, but this has not been performed. More often, one kind dominates the departures from normal scattering, whereby this approximation suffices to initiate a fuller analysis. Then, analyzed in terms of the observable intensities *I*(**h**) = *K*|^λ^ *F*(**h**)|^2^,

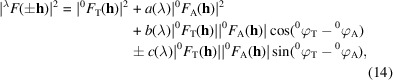

where *a*(λ) = (|*f* ^Δ^|/*f* ^0^)^2^, *b*(λ) = 2(*f*′/*f* ^0^) and *c*(λ) = 2(*f*″/*f* ^0^).

The coefficients *a*(λ), *b*(λ) and *c*(λ) specify the spectral dependences of the anomalous scattering factors, and |^0^ *F*_T_|, |^0^ *F*_A_| and Δφ = ^0^φ_T_ − ^0^φ_A_ are the wavelength-invariant contributions from Thomson scattering and relate directly to electron density.

The formulation of (14) also provides the basis for understanding the information content in the MAD experiment. Without approximation, the intensity differences between Friedel mates or their symmetry equivalents are

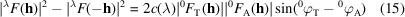

and, in the typical situation where anomalous scattering is weak relative to normal scattering |^0^ *F*_T_(**h**)| ≃ ½[|^λ^F(**h**)| + |^λ^F(−**h**)|]. This gives the Bijvoet difference:

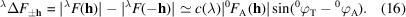

Similarly, a measure of phase information coming from intensity variation with wavelength, the anomalous dispersion, derives from equation (14). Taking 〈|^λ^ *F*(**h**)|^2^〉 = ½[|^λ^ *F*(**h**)|^2^ + |^λ^ *F*(−**h**)|^2^], again without approximation, the difference in intensities at two selected wavelengths λ_*i*_ and λ_*j*_ is

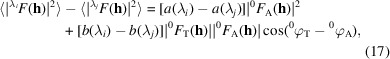

and, unless anomalous scattering is very strong, the second term greatly dominates; moreover, |^0^ *F*_T_(**h**)| ≃ 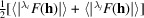 . This then gives the dispersive difference,

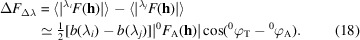

Comparing equations (16) and (18), one sees that Bijvoet differences are proportional to *f*′′(λ) and to sin(Δφ), while dispersive differences are proportional to |*f*′(λ_*i*_) − *f*′(λ_*j*_)| and to cos(Δφ), which demonstrates the orthogonality of information needed for definitive phase evaluation.

The Bijvoet and dispersive differences given by equations (16) and (18) also serve as the basis for estimating the expected ratio of averaged anomalous diffraction to averaged total diffraction. Ratios are obtained for the root-mean-squared (r.m.s.) values of the respective diffraction differences relative to that of the total normal diffraction (Hendrickson, 1991 ▸),

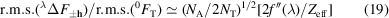

and

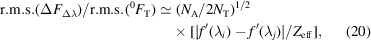

where *N*_A_ is the number of anomalous scatterers with scattering factors *f*′ and *f*′′, *N*_T_ is the number of non-H protein atoms and *Z*_eff_ is the effective atomic number for an average non-H atom. For typical protein molecules, *Z*_eff_ = 6.7.

## 11. Scattering-factor evaluation for MAD optimization

**Figure 5:**
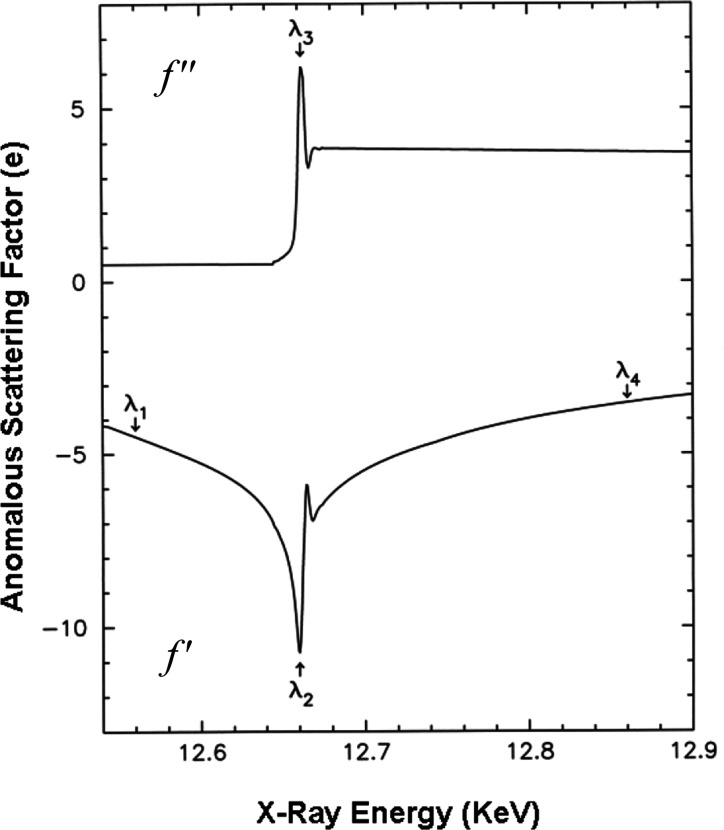
Anomalous scattering-factor spectra at the Se *K* edge of selenomethionyl human chorionic gonadotropin, derived from fluorescence measurement from a crystal as fitted with Kramers–Kronig transformation to theoretical selenium factors outside the range 12.648–12.676 keV. Adapted from Fig. 11 of Wu *et al.* (1994 ▸) with permission from Elsevier.

Anomalous scattering factors, which are needed for the design and analysis of MAD experiments, can be evaluated from fluorescence spectra measured from the very crystal used for diffraction measurements. The normal, Thomson scattering factors are obtained by transformation from quantum calculations of radial electron-density distributions. Experimental evaluation of anomalous scattering is important, however, because resonant features depend on transitions to unoccupied orbitals, which are critically affected by details of the molecular environment. Quantum calculations (Cromer & Liberman, 1970 ▸) for the isolated atoms provide a baseline for designs; however, the molecular reality is often appreciably different near the absorption edge itself. The calculated values remain valid at positions away from the edge, and we standardly normalize both the *f*′′ spectrum and the Kramers–Kronig-derived *f*′ spectrum to the theoretical spectra, splicing experimental near-edge features into the theoretical framework (Hendrickson *et al.*, 1988 ▸; Evans & Pettifer, 2001 ▸). Such spectra are shown in Fig. 5 ▸ for selenium from a selenomethionyl protein.

While full optimization of a MAD experiment depends on varied considerations, extrema from the spectra usually dictate the wavelengths of choice. From the Bijvoet-difference dependence of equation (16), one clearly seeks the wavelength of maximal *f*′′ (λ_3_ in Fig. 5 ▸), and from the dispersive difference dependence of equation (18) and knowledge of the theoretical form of the *f*′ spectrum one chooses the wavelength of minimal *f*′ (λ_2_ in Fig. 5 ▸) for contrast with one or more remote wavelengths (λ_1_ and λ_4_ in Fig. 5 ▸). Note that the *f*′ spectrum is roughly the negative of the first derivative of the *f*′′ spectrum. Thus, the *f*′ minimum is at the rising inflection point of the *f*′′ spectrum and there is a local *f*′ maximum at the descending inflection point.

**Figure 6:**
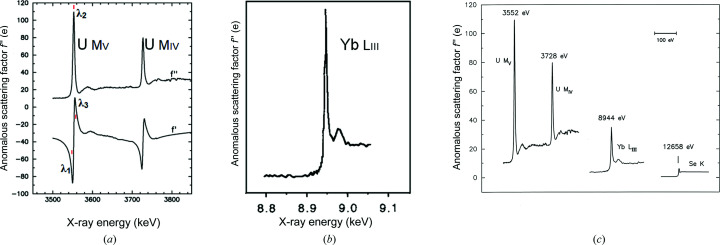
Comparison of sharp anomalous scattering spectra at *M*, *L* and *K* edges. (*a*) Resonance features at the *M*_V_ and *M*_IV_ absorption edges of uranyl nitrate (Liu *et al.*, 2001 ▸). Wavelength positions at the *M*_V_ edge are marked by red lines. (*b*) Spectrum of the *f*′′ component of Yb *L*_III_ anomalous scattering from ytterbium-derivatized N-cadherin D1 (Shapiro *et al.*, 1995 ▸). (*c*) Direct at-scale comparison of the U *M*_V_ edge, Y *L*_III_ edge and Se *K* edge in Figs. 6 ▸(*a*), 6 ▸(*b*) and 5 ▸, respectively. The U *M*_IV_, Yb *L*_III_ and Se *K* edge energies are on a linear scale of X-ray energy. (*a*) and (*c*) are reproduced from Liu *et al.* (2001 ▸) with permission from the National Academy of Sciences. Copyright (2001) National Academy of Sciences. (*b*) is reproduced from Shapiro *et al.* (1995 ▸) with permission from Springer Nature.

Features in anomalous scattering spectra vary widely among elements and absorption edges. Some are nearly featureless (notably mercury) and others are incredibly sharp and strong. Fig. 6 ▸(*a*) shows the sharp features of uranium spectra. The Bijvoet scattering strength at the *M*_V_ peak of *f*′′ (λ_2_) is 105 electrons, and the dispersive scattering difference |*f*′(λ_3_) − *f*′ (λ_1_)| between values at the descending *f*′′ inflection point (λ_3_) and the rising *f*′′ inflection point (λ_1_) is 100 electrons, over a peak width of only 5 eV. Low-energy X-ray diffraction experiments are challenging; nevertheless, we did accomplish a U *M*_IV_-edge MAD structure test on uranyl elastase at 3.2 Å resolution (Liu *et al.*, 2001 ▸). Fig. 6 ▸(*b*) shows the *f*′′ spectrum at the Y *L*_III_ edge from a complex with N-cadherin, which has a Bijvoet-peak scattering strength of 30.2 electrons (Shapiro *et al.*, 1995 ▸). The anomalous scattering strength increases in general from *K* to *L* to *M* edges, as seen in the at-scale comparison of *f*′′ spectra in Fig. 6 ▸(*c*), and variably so depending on bonding and coordination interactions.

In modern practice, although fluorescence spectra are used to define the maxima and inflection points for wavelength selections, the actual anomalous scattering factors themselves are quantified by refinement, following earlier practice (Templeton & Templeton, 1982 ▸; Fanchon & Hendrickson, 1990 ▸; Weis *et al.*, 1991 ▸). It is even possible to perform site-specific scattering-factor refinements to yield spectra from which redox states can be deduced (Spatzal *et al.*, 2016 ▸).

## 12. Implementation of MAD phasing

**Figure 7:**
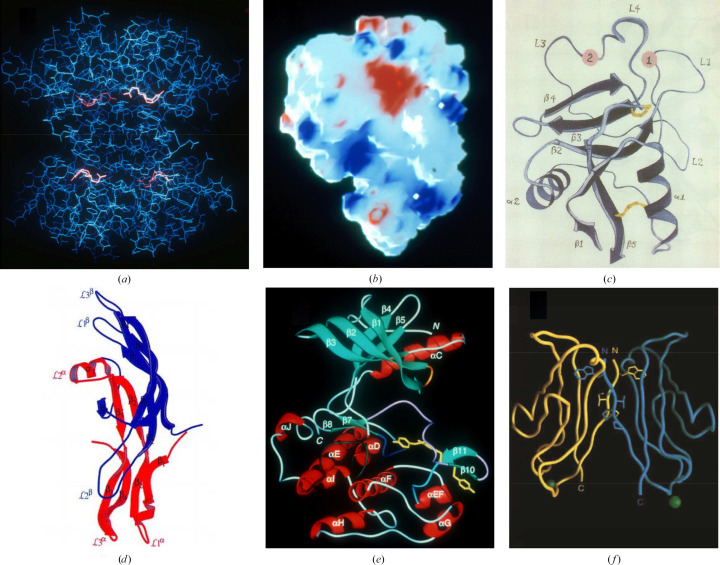
Molecular drawings of selected early MAD-phased structures. (*a*) Streptavidin complexed with selenobiotin (Hendrickson *et al.*, 1989 ▸). (*b*) Selenomethionyl ribonuclease H from *E. coli* (Yang *et al.*, 1990 ▸). (*c*) Lectin domain from rat mannose-binding protein (Weis *et al.*, 1991 ▸). (*d*) Human glycoprotein hormone chorionic gonadotropin (Wu *et al.*, 1994 ▸). (*e*) Tyrosine kinase domain of the human insulin receptor (Hubbard *et al.*, 1994 ▸). (*f*) Adhesive domain CD1 of murine N-cadherin (Shapiro *et al.*, 1995 ▸). Adapted with permission from AAAS (*b*) and (*c*), Elsevier (*d*) and Springer Nature (*e*, *f*).

With our MAD formulation (equation 14) in hand and given promising diffraction estimates (equations 19 and 20), we validated MAD in four-wavelength tests at the Fe *K* edge of lamprey hemoglobin: first at 5.5 Å resolution (Hendrickson, 1985 ▸) and then at 3.0 Å resolution (Hendrickson *et al.*, 1988 ▸). Meanwhile, independent MAD experiments were also reported (Kahn *et al.*, 1985 ▸; Guss *et al.*, 1988 ▸). We then used and further developed MAD in solving the structures of several significant biomolecular structures (Hendrickson *et al.*, 1989 ▸; Yang *et al.*, 1990 ▸; Ryu *et al.*, 1990 ▸; Weis *et al.*, 1991 ▸; Leahy *et al.*, 1992 ▸; Wu *et al.*, 1994 ▸; Hubbard *et al.*, 1994 ▸; Shapiro *et al.*, 1995 ▸, 1996 ▸; McDonald *et al.*, 1995 ▸; Georgiadis *et al.*, 1995 ▸; Athappilly & Hendrickson, 1995 ▸; Peat *et al.*, 1996 ▸; Zhu *et al.*, 1996 ▸; Lima *et al.*, 1997 ▸; Yamaguchi & Hendrickson, 1996 ▸; Cuff *et al.*, 1998 ▸; DiGabriele *et al.*, 1998 ▸). Six of our first MAD structures are shown in Fig. 7 ▸. Worldwide adoption of MAD also grew dramatically during this period (Hendrickson, 2014 ▸).

**Figure 8:**
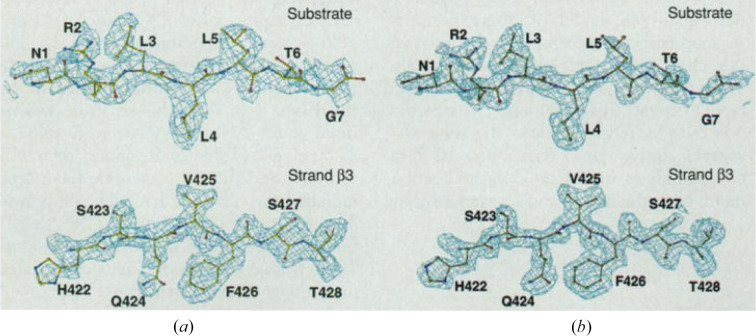
Validation of the accuracy of MAD-phased electron density. Two segments are shown from electron-density maps of the DnaK SBD (Zhu *et al.*, 1996 ▸): a bound substrate peptide (top) and strand β3 (bottom). The direct experimental map at 2.3 Å resolution (no density modification) is shown in (*a*) and the 2*F*_o_ − *F*_c_ map after refinement at 2.0 Å resolution is shown in (*b*). The refined atomic model is superimposed onto the maps in both (*a*) and (*b*). Reproduced from Zhu *et al.* (1996 ▸) with permission from AAAS.

From the outset, the aspiration for MAD phasing was for the resolution of the phase ambiguity that had vexed SAD and SIR experiments. This indeed was accomplished. For example, Fig. 8 ▸ illustrates the degree of perfection in resulting experimental electron-density maps from our MAD structure of the substrate-binding domain (SBD) of selenomethionyl DnaK compared with those from the refined atomic model.

Three technical developments contributed especially strongly to the efficacy and explosive growth of MAD phasing: MAD-adapted synchrotron beamlines, selenomethionyl proteins and cryoprotection. Initially, we performed experiments at various synchrotrons around the world, improvising strategies and employing varied detector systems, we exploited several different anomalous elements, introducing them in various ways, and we measured data from crystals mounted in glass capillaries, either at room temperature or cooled slightly to ∼0°C. Later, we and others used beamlines specifically designed or adapted for MAD, commonly incorporated selenomethionine through recombinant protein expression, and universally adopted cryoprotection to ∼100 K to control radiation damage.

A MAD beamline needs to provide fine control of the X-ray energy at any of many potential absorption edges, precise goniometry for crystal positioning, and a suitable diffraction detector system. We designed and built beamline X4A at NSLS expressly for our MAD experiments (Staudenmann *et al.*, 1989 ▸), and other similarly purposed early beamlines (for example 5.0.2 at ALS, BM14 at ESRF and SBC-CAT 19ID at APS) also accommodated the growing demand for MAD. Somewhat later, the fixed-wavelength NE-CAT 24ID-E beamline at APS was set up to address Se SAD.

Although the metalloproteins and heavy-atom derivatives used for MIR are excellent sources for MAD phasing, a more general phasing vehicle is clearly desirable. Selenomethionine is just such an agent. We had used selenolanthionine in our initial MAD trials (Hendrickson, 1985 ▸), and we synthesized selenobiotin for our initial novel MAD structure (Hendrickson *et al.*, 1989 ▸); hence, appreciating the potential benefit from the analogous replacement for methionine in proteins, we set out to produce selenomethioninyl proteins. We found that Cowie & Cohen (1957 ▸), studying translation, had already done so in *Escherichia coli*, and we confirmed that *E. coli* cultures could be maintained when supplied exclusively with selenomethionine, meaning that recombinant proteins could have 100% replacement of selenium for sulfur in their methionines (Hendrickson *et al.*, 1990 ▸). Selenium incorporation also proved to be possible in mammalian cell cultures (Wu *et al.*, 1994 ▸), although usually not at the 100% level. Selenomethionyl proteins are widely used for MAD and SAD phasing (Hendrickson, 2014 ▸).

MAD experiments are demanding in that one seeks to obtain compatible data sets at three or four wavelengths, which is a serious challenge because of radiation damage. To the good fortune of MAD experiments, techniques for the cryopreservation of macromolecular crystals in vitreous ice were innovated (Hope, 1988 ▸) and perfected (Rodgers, 1994 ▸) contemporaneously with the introduction of MAD. The rate of radiation damage is reduced ∼50-fold at 100 K compared with 300 K (Warkentin & Thorne, 2010 ▸). Our cryoprotected MAD structure of human chorionic gonadotropin (Wu *et al.*, 1994 ▸) was our first of what became the standard for MAD and SAD diffraction studies.

With these critical developments in place, we applied MAD (or occasionally SAD) phasing to many additional biological structure problems, primarily using the combination of beamline X4A, selenomethionyl proteins and cryoprotected crystals (Jiang *et al.*, 2000 ▸; Stauber *et al.*, 2000 ▸; Marina *et al.*, 2001 ▸, 2005 ▸; Min *et al.*, 2003 ▸; Kovall & Hendrickson, 2004 ▸; Williams, Sue *et al.*, 2005 ▸; Williams, Xie *et al.*, 2005 ▸; Liu & Hendrickson, 2007 ▸; Martinez-Hackert & Hendrickson, 2007 ▸, 2009 ▸, 2011 ▸; Williams *et al.*, 2007 ▸; Cheung *et al.*, 2008 ▸, 2009 ▸; Cheung & Hendrickson, 2008 ▸, 2009 ▸; Moore & Hendrickson, 2009 ▸, 2012 ▸; Zhang & Hendrickson, 2010 ▸; Chen *et al.*, 2010 ▸; Collins & Hendrickson, 2011 ▸; Qi *et al.*, 2013 ▸; Chang *et al.*, 2014 ▸; Zhang *et al.*, 2014 ▸; Yang *et al.*, 2014 ▸; Guo *et al.*, 2015 ▸; Assur Sanghai *et al.*, 2018 ▸; Gong *et al.*, 2023 ▸).

## 13. Alternative approaches in MAD phasing analysis

Our theoretical analysis of MAD (equation 14) describes the set of observed intensities for the Bijvoet mates at various wavelengths, {|^λ^ *F*(±**h**)|^2^}, in terms that separate wavelength-variant factors [*a*(λ), *b*(λ) and *c*(λ)] from factors involving the wavelength-invariant structure-factor quantities |^0^ *F*_T_(**h**)|, |^0^ *F*_A_(**h**)| and Δφ = (^0^φ_T_ − ^0^φ_A_). We devised a procedure for solving this system of equations for each reflection **h** subject to trigonometric identities. The set of {|^0^ *F*_A_(**h**)|} structure factors serves to define the atomic substructure of anomalous scattering centers, by Patterson or direct methods, and then to refine this substructure, subject to enantiomorph ambiguity. Phases calculated from the substructure then solve the phase problem, ^0^φ_T_ = 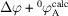 , provided that the substructure {±**r**_A_} is taken in the correct handedness.

**Figure 9:**
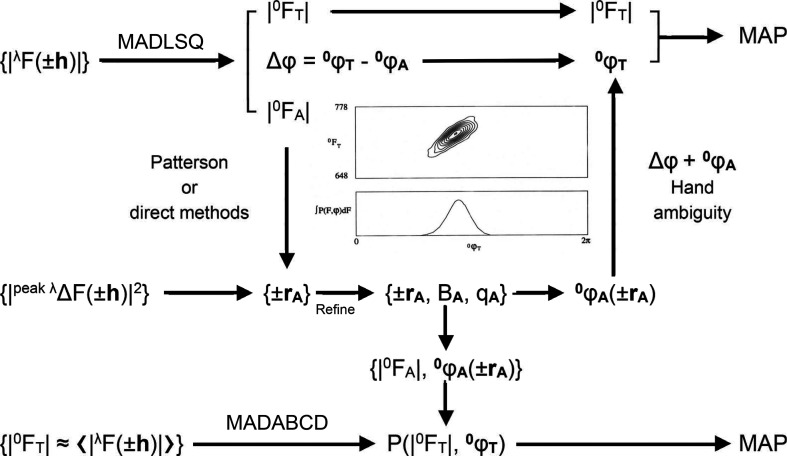
Schematic diagram of phase evaluation from MAD data, Bijvoet mates at multiple wavelengths {|^λ^ *F*(±**h**)|^2^}, by two alternative approaches. Algebraic analysis by *MADLSQ* deduces |^0^ *F*_T_|, |^0^ *F*_A_| and Δφ = ^0^φ_T_ − ^0^φ_A_ for each reflection, from which {|^0^ *F*_A_|} generates the substructure of anomalous scatterers {±**r**_A_}. When taken in the correct hand, this solves the phase problem. In the phase probability pathway of *MADABCD*, the average of {|^λ^ *F*(±**h**)|^2^} provides an estimate for |^0^ *F*_T_|, the peak Bijvoet differences {^λ^Δ*F*_±**h**_} yield {±**r**_A_}, and these observations form the basis for computing the joint probability P(|^0^ *F*_T_|, ^0^φ_A_|), an example of which is shown in the inset.

The steps of this algebraic approach to MAD analysis, embodied in the program *MADLSQ*, are schematized in Fig. 9 ▸. The effectiveness of the process is epitomized by results from MAD phasing for the SBD of the molecular chaperone DnaK (Zhu *et al.*, 1996 ▸). This structure was refined at 2.0 Å resolution after model building based on a 2.3 Å resolution electron-density map, which in turn was derived from a substructure determined at 3.0 Å resolution. We adopted a ‘phase first, merge later’ strategy in *MADLSQ*, whereby replication statistics define the precision of the analysis. Here, the replicates at 2.3 Å resolution gave merging statistics of *R*(|^0^ *F*_T_|) = 0.051, *R*(|^0^ *F*_A_|) = 0.356, 〈Δ(Δφ)〉 = 36.5° and 〈σ(Δφ)〉 = 17.2°, where *R* = 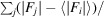 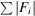 for replicates *i* within each unique reflection *j*, Δ(Δφ) is the discrepancy between Δφ estimates from a pair of replicates and σ(Δφ) is the estimate of the Δφ error from the least-squares minimization. *R*(|^0^ *F*_T_|) is commensurate with the *R*_sym_ value of 0.052, the higher value for *R*(|^0^ *F*_A_|) is compatible with expectation from the partial structure contribution of the six Se atoms to the total diffraction, and the precision of phase evaluations is consistent with the comparability of the experimental and the refined electron-density maps (Fig. 8 ▸).

A phase probability approach based on the factorized formalism of equation (14) provides a practical alternative to the algebraic approach of *MADLSQ*. This approach circumvents the defect that the least-squares analysis requires a minimal set of data for each reflection, whereas phase information often still exists when only a subset is available. A probability analysis based on equation (14) provides a comprehensive description of the phase information from anomalous diffraction (Pähler *et al.*, 1990 ▸). In our *MADABCD* reformulation of the procedure, also illustrated in Fig. 9 ▸, we first estimate |^0^ *F*_T_| from the wavelength-dependent observations |^0^ *F*_T_| ≃ 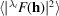 ; next, recognizing experience from numerous SAD analyses showing that peak Bijvoet differences (^λ^Δ*F*_±**h**_ in equation 16) suffice for substructure determinations, we calculate |^0^ *F*_A_| and ^0^φ_A_ from the refined substructure {**r**_A_}, and finally we produce the joint probability distributions, *P*(|^0^ *F*_T_|, ^0^φ_T_), which upon integration yield the best Fourier coefficients. Through integration over all possible values of |^0^ *F*_T_| in this two-dimensional probability function, a phase probability, *P*(^0^φ_T_), consistent with all available data is obtained (Fig. 9 ▸), and similarly for *P*(|^0^ *F*_T_|).

A third alternative can be called the MAD-as-MIR approach, akin to the suggestions of Phillips & Hodgson (1980 ▸). A MAD experiment can be considered to be an *in situ* MIR experiment where differences are realized from physics instead of from chemistry. The dispersive differences (equation 18) are like those of MIR differences and the Bijvoet differences (equation 16) are like the anomalous differences that are included to give MIRAS. In the MAD-as-MIR approach, the data set at one of the wavelengths is chosen arbitrarily (also rather unsatisfyingly) as the reference (*F*_P_) data set, and differences are taken relative to these data. Ramakrishnan *et al.* (1993 ▸) analyzed the structure of histone H5 using both *MADLSQ* and a MAD-as-MIR approach that employed *MLPHARE* (Otwinowski, 1991 ▸). They reported that the latter ‘gave somewhat better maps than the algebraic formalism’, and this method of analysis quickly took hold (Ramakrishnan & Biou, 1997 ▸).

**Figure 10:**
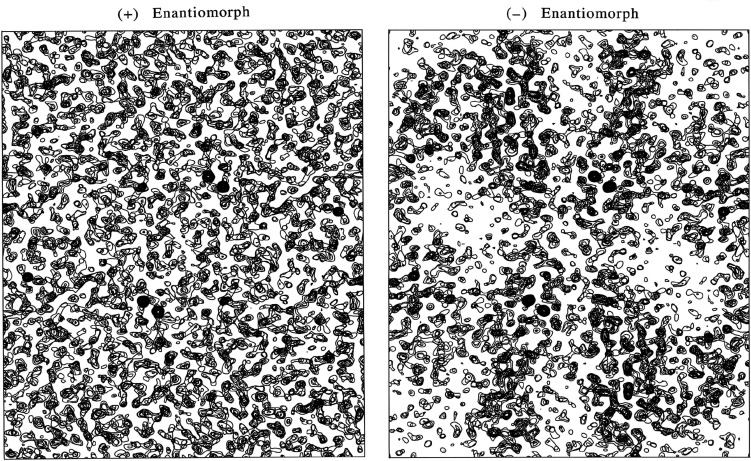
Enantiomorph definition. Alternative electron-density maps at 2.3 Å resolution are shown from the analysis of the DnaK SBD (Zhu *et al.*, 1996 ▸). One is based on phases from the substructure {+**r**_A_}, as deduced by Patterson analysis from {|^0^ *F*_A_|} values, and the other is based on the alternative {−**r**_A_} where the *z* coordinates of the projection are reversed and equivalent slabs are shown. Interpretable protein features and appropriately featureless solvent expanses characterize the correct alternative on the right.

It is worth emphasizing that anomalous diffraction defines absolute configuration (Bijvoet *et al.*, 1951 ▸), while conventional diffraction analysis is insensitive to handedness. Thus, as mentioned above, the substructure of anomalous scatterers is unavoidably ambiguous {±**r**_A_}, it being the case that the set of {**r**_A_} positions and its enantiomorphic set {−**r**_A_} equally satisfy the |^0^ *F*_A_| or |^λ^Δ*F*_±**h**_| coefficients. On the other hand, only the correct enantiomer will be compatible with the signs as well as the magnitudes of the Bijvoet differences ^λ^Δ*F*_±**h**_. A statistical basis for resolving the enantiomorph ambiguity has proved to be elusive, but electron-density maps derived from the alternatives are decisive when phase evaluations are accurate. The density map for DnaK SBD in the correct hand [Fig. 10 ▸(*b*)] shows clear-cut molecular boundaries and structural features, whereas the map from the wrong hand [Fig. 10 ▸(*a*)] contains no recognizable features.

## 14. Single-wavelength anomalous diffraction (SAD) and density modification

SAD is a subset of MAD, and both the theoretical formalism and many aspects of the analytical procedures also apply for SAD as for MAD. The intrinsic phase definition from SAD is sharp but, with two equally likely alternatives for each reflection, the density-map features become blurred. The orthogonality of dispersive contributions in MAD readily resolves this ambiguity; however, MAD is not always an option. The absorption edges of lighter elements (*Z* < 26; for example phosphorus, sulfur, potassium, calcium and manganese) and some intermediate elements (for example iodine and xenon) are inaccessible at most synchrotron beamlines (Hendrickson & Ogata, 1997 ▸) and, in any case, low-energy diffraction experiments are complicated by high absorption and large Bragg angles. Moreover, a substructure of light atoms is relatively feeble as a partial structure for resolving the phase ambiguity, as we found with the S atoms of crambin (Hendrickson & Teeter, 1981 ▸).

A hint into ambiguity resolution was already evident from our analysis of trimeric hemerythrin. Being pre-MAD, we used Cu *K*α radiation to exploit the anomalous scattering from the dimeric iron center in hemerythrin (Smith *et al.*, 1983 ▸) as ‘resolved’ by the iron partial structure. The initial map was inadequate for interpretation on its own; however, threefold molecular averaging and ‘solvent leveling’, as it was then described, improved the map dramatically. A similarly dramatic enhancement from threefold symmetry averaging had been seen in analyzing the structure of influenza virus haemagglutinin (Wilson *et al.*, 1981 ▸), which used procedures devised by Bricogne (1976 ▸) and applied decisively in studies with 17-fold (Champness *et al.*, 1976 ▸) and 5-fold (Winkler *et al.*, 1977 ▸) averaging. Although noncrystallographic symmetry is not always present, fluid solvent expanses are universal features of macromolecular crystals.

Being fluid, or cryo-vitrified, these solvent regions have a featureless, constant electron density, and, in an early article on phase evaluation, I described a procedure for using solvent constancy in phase refinement through iterative Fourier cycling such as used in molecular averaging (Hendrickson, 1981 ▸). We had used this procedure in our analyses of low-resolution structures of myohemerythrin (Hendrickson *et al.*, 1975 ▸) and octameric hemerythrin (Ward *et al.*, 1975 ▸) as well as in tests with orthorhombic *Glycera* hemoglobin (Hendrickson, 1981 ▸). Wang (1985 ▸) innovated general and powerful procedures for molecular envelope definition and solvent flattening, and, as applied to anomalous scattering problems, it was called the iterative single-wavelength anomalous scattering (ISAS) method. Successes followed for a neurophysin–dipeptide complex using an iodinated derivative (Chen *et al.*, 1991 ▸) and in the corrected analysis of Cd,Zn metallothionein (Robbins *et al.*, 1991 ▸).

Density modification advanced beyond Wang’s introduction of solvent flattening (Wang, 1985 ▸) with the incorporation of histogram matching (Zhang & Main, 1990 ▸), the systematic inclusion of molecular averaging (Cowtan & Main, 1993 ▸), the innovation of solvent flipping (Abrahams & Leslie, 1996 ▸) and the introduction of maximum-likelihood processing (Terwilliger, 2000 ▸). As implemented in programs such as *DM* and reviewed by Cowtan (2010 ▸), density modification became a mainstay for phase refinement and map improvement.

**Figure 11:**
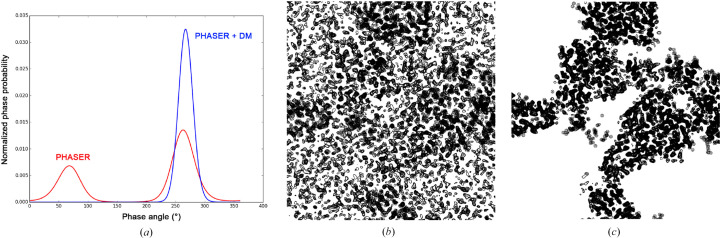
SAD ambiguity resolution from density modification. Examples are taken from the sulfur SAD analysis of DnaK–ATP (Liu *et al.*, 2013 ▸). (*a*) Phase probability distributions for a particular reflection as evaluated in *Phaser* from SAD combined with the S-atom partial structure (red) and after using *DM* for density modification that excluded molecular averaging. (*b*) A portion of the electron-density distribution phased from SAD combined with the sulfur partial structure. (*c*) The same portion of the map after density modification.

The results for ambiguity resolution in SAD phasing are dramatic. This is well illustrated in our sulfur-SAD analysis of DnaK–ATP (Liu *et al.*, 2013 ▸). A typical probability distribution shows essentially perfect phase resolution [Fig. 11 ▸(*a*)] and, overall, the density-modified map [Fig. 11 ▸(*c*)] is strikingly improved over the SAD map [Fig. 11 ▸(*b*)]. This SAD map was produced with *Phaser* (Read & McCoy, 2011 ▸), including partial structure information from the sulfur substructure and, to make this a generally applicable test, *DM* was performed without using the noncrystallographic symmetry that happened to exist. Map quality was judged by the map correlation coefficient (MapCC), which compares a given map with that from the ultimate refined atomic model. MapCC improved from 46.6% for the SAD map to 85.3% for the *DM*-modified map, from which 1117 of the 1200 residues (93%) in this DnaK–ATP structure were built automatically by the *ARP*/*wARP* program (Perrakis *et al.*, 1999 ▸).

## 15. Evolution from MAD to SAD

It took some time for the power of density-modified SAD phasing to be recognized, and even for the acronym SAD to take root. In part, the success of MAD obviated the need for an alternative. After crambin (Hendrickson & Teeter, 1981 ▸), the next single-wavelength anomalous applications were those for B.-C. Wang’s ISAS studies (Chen *et al.*, 1991 ▸; Robbins *et al.*, 1991 ▸), and for a lead derivative of a translation-factor domain (Biou *et al.*, 1995 ▸) and a selenomethionyl enzyme (Turner *et al.*, 1998 ▸). ‘SAD’ was used in describing experiments for a GFP structure (Ormö *et al.*, 1996 ▸), for systematic methodology tests (Dauter *et al.*, 1999 ▸; Dauter & Dauter, 1999 ▸) and for the structure of psoriasin (Brodersen *et al.*, 2000 ▸). Influentially, a re-evaluation of seven MAD structures of selenomethionyl proteins suggested that density-modified SAD maps rivaled MAD maps or could even rescue failed MAD analyses (Rice *et al.*, 2000 ▸). The *Jolly SAD* review by Dauter *et al.* (2002 ▸), presenting 15 tests and novel applications, heralded the start of the SAD era.

**Figure 12:**
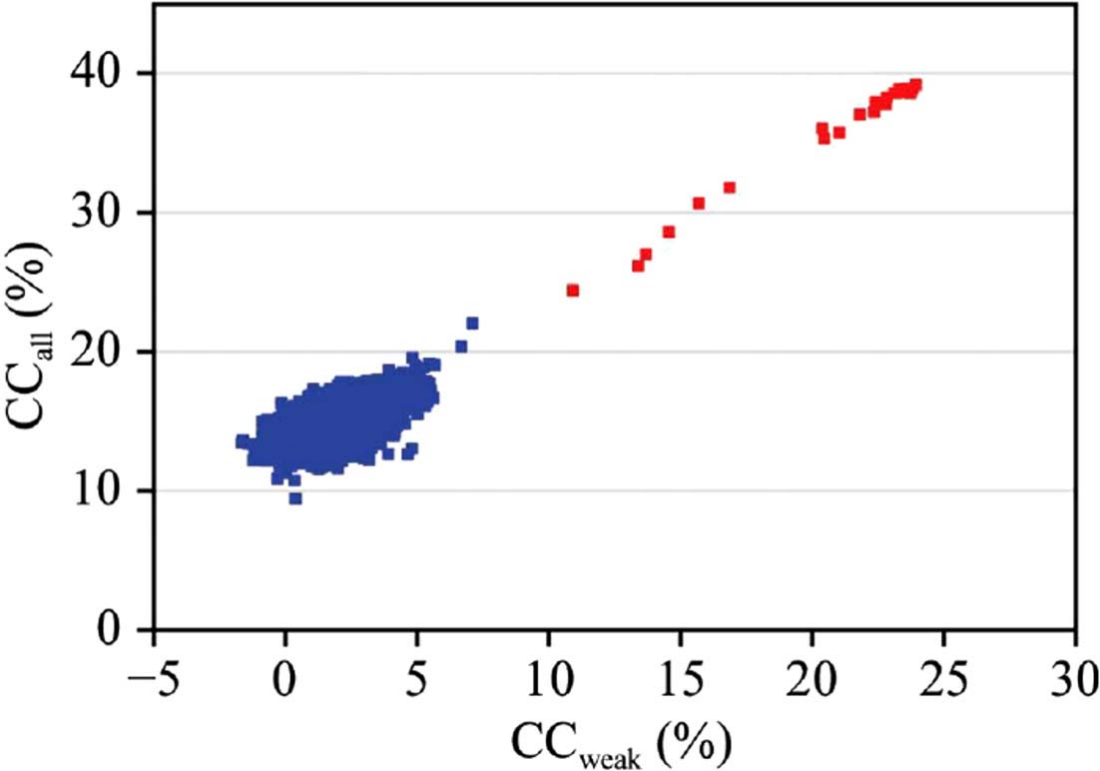
Profile of correlation coefficients (CCs) from *SHELXD* for the sulfur substructure in the SAD analysis of DnaK–ATP (Liu *et al.*, 2013 ▸). The distribution of CC_all_ versus CC_weak_ values is shown for 10 000 trials; the 36 successful solutions are colored red and the 99.6% random results are shown in blue. Reproduced from Fig. 5(*f*) of Liu *et al.* (2013 ▸).

Structures of ever-increasing size came under study, and increasingly these investigations were on selenomethionyl proteins. Consequently, the sizes of selenium substructures increased with sequence length in proportion to the methionine frequency, ∼1:59. An advance of comparable importance to density modification came in using direct methods for substructure determinations. Bijvoet differences (equation 16), typically taken at the wavelength of peak *f*′′, are converted to normalized structure-factor estimates, |*E*_A_| = 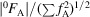 , for the substructure and used in direct-methods programs. Turner *et al.* (1998 ▸) implemented this idea, adopting the *Shake-and-Bake* (*SnB*) dual-space recycling procedure (Miller *et al.*, 1994 ▸; Smith *et al.*, 1998 ▸) to find 30 selenium sites. Many other selenomethionyl-protein applications soon followed (Deacon & Ealick, 1999 ▸), including a 70-selenium enzyme (Deacon *et al.*, 2000 ▸). Schneider & Sheldrick (2002 ▸) employed an efficient tangent-formula-based version of *SnB* in *SHELXD*, and a typical result is shown in Fig. 12 ▸ from our native SAD application to DnaK–ATP (Liu *et al.*, 2013 ▸). Here, a few (red) among 10 000 random trials stand out from random in the plot of CC_all_ versus CC_weak_, each an equivalent solution for this 52-atom anomalous substructure.

Among the many selenomethionyl structures solved by SAD, particularly impressive examples come from large and challenging protein complexes produced from mammalian cell cultures and analyzed at modest resolution. The first structure of RAG1/RAG2 recombinase identified 52 of 58 selenium sites at 3.7 Å resolution (Kim *et al.*, 2015 ▸) and the first structural analysis of DNAPKcs identified 172 of 236 selenium sites at 4.3 Å resolution (Sibanda *et al.*, 2017 ▸). The Se sites for RAG1/RAG2 were found by *SHELXD*, but those for DNAPKcs used maps that were based on a 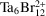 cluster derivative.

## 16. Native SAD

Structural analysis from native molecules, without recourse to any modification, is an aspiration for structural biologists, and advances in SAD phasing now make native SAD accessible for the vast majority of macromolecules (Liu *et al.*, 2012 ▸; Weinert *et al.*, 2015 ▸). The crambin structure certainly qualifies as native SAD, although it was not so named at the time; however, the crambin structure is atypical in being quite small (46 residues) and at exceptional resolution [initially 1.5 Å (Hendrickson & Teeter, 1981 ▸) and later 0.48 Å (Schmidt *et al.*, 2011 ▸)]. Sulfur SAD as resolved by solvent flattening was brought into play by Liu *et al.* (2000 ▸). Suggestions emerged that native SAD phasing could routinely be based on the S atoms of proteins (Dauter *et al.*, 1999 ▸) and the P atoms of nucleic acids (Dauter & Adamiak, 2001 ▸) and that the use of lower X-ray energy (longer wavelength) would improve effectiveness (Weiss *et al.*, 2001 ▸; Yang *et al.*, 2003 ▸; Wagner *et al.*, 2016 ▸). Experiments at lower energy do present complications, however.

**Figure 13:**
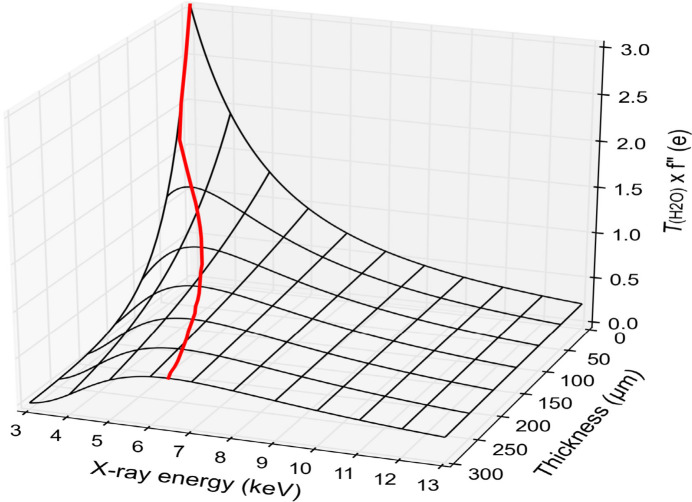
Dependence of transmitted anomalous signals on crystal thickness and X-ray energy. Water is taken to approximate the absorptivity of a typical macromolecular crystal, and we plot the transmitted *f*′′ anomalous signal from sulfur as a function of energy and thickness. The red line shows the ridge of maximal signal in this parameter space. This presentation is adapted from data presented in Fig. 2 of Liu *et al.* (2013 ▸).

The absorption edges for P (2.14 keV) and S (2.47 keV) are remote from the energies that can be reached by most beamlines. Moreover, as the X-ray energy is lowered to increase *f*′′ for a low-*Z* element, difficulties arise for detector geometry from the consequent large scattering angles, for crystal thickness from an increased absorption coefficient and for background from pathway scatter. As shown in Fig. 13 ▸, above a critical thickness the signal as actually transmitted deteriorates with the lowering of X-ray energy. Microcrystals solve the thickness problem, but then radiation damage limits the crystal lifetime; the merging of data from multiple crystals addresses radiation damage and can also improve statistics by achieving higher data multiplicity (Liu *et al.*, 2012 ▸). Measurements at multiple orientations also increase accuracy from multiplicity (Weinert *et al.*, 2015 ▸). Pathway absorption and scattering at lower energy can be addressed by operation in vacuum (Aurelius *et al.*, 2017 ▸) or in a helium environment (Karasawa *et al.*, 2022 ▸).

**Figure 14:**
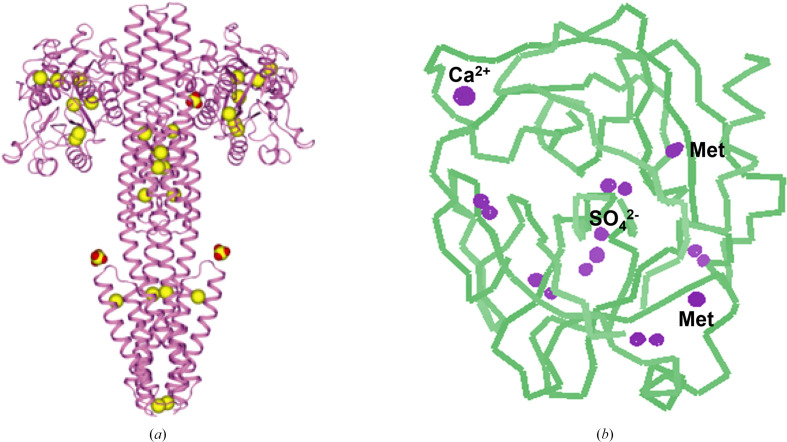
Sulfur substructures from native SAD analyses. (*a*) Ribbon diagram of the TorT–TorS_S_ ligand–histidine kinase complex showing the 28 S atoms (yellow) and three sulfate ions (yellow with red O atoms) used to define 1148 ordered protein residues. This structural analysis was performed at 7 keV [*f*′′(S) = 0.73 electrons]. (*b*) C^α^ backbone model of bovine trypsin showing the 16 peaks above 6σ in a Bijvoet difference map. A calcium ion has a peak height of 47σ and the sulfur peaks range from 9.6σ to 18.9σ. The peaks from calcium (Ca^2+^) and sulfate ( 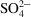 ) ions and from two methionine S atoms (Met) are labeled. The other 12 peaks are in disulfide-bridged pairs. This structural analysis was performed at 12.7 keV [*f*′′(S) = 0.235 electrons]. (*a*) was adapted from Fig. 9(*d*) of Liu *et al.* (2013 ▸).

Even just from the five examples of our initial multi-crystal sulfur SAD experiments (Liu *et al.*, 2012 ▸), we estimated that ∼90% of then-current PDB depositions were within the limits of these examples: fewer than 1200 residues, fewer than 32 sulfur sites and better than 2.8 Å resolution [Fig. 14 ▸(*a*)]. Since that time, native SAD has indeed entered the mainstream (Rose *et al.*, 2015 ▸; Liu & Hendrickson, 2015 ▸). Five more-recent applications (El Omari *et al.*, 2014 ▸; Akey *et al.*, 2016 ▸; Zeng *et al.*, 2019 ▸; Basu *et al.*, 2019 ▸) are all at resolutions worse than the previous 2.8 Å limit (3.4–2.95 Å when refined; often much lower for phasing) and extend the size limit to 2362 residues, of which 118 are sulfur-containing. Sulfur SAD determinations have also been reported from FEL experiments by serial femto­second crystallography (Nass *et al.*, 2020 ▸). Although many native SAD efforts have used low X-ray energy (long wavelength) to increase the anomalous scattering strength (Basu *et al.*, 2019 ▸; Nass *et al.*, 2020 ▸; Karasawa *et al.*, 2022 ▸) even the sulfur signal at the Se *K* edge (*f*′′ = 0.235 electrons) can suffice. Thus, we now use sulfur SAD phasing of trypsin at the Se *K* edge to test the performance of the NYX beamline [Fig. 14 ▸(*b*)].

## 17. Anomalous diffraction and structural genomics

As MAD and SAD were advancing, DNA-sequencing efforts were advancing ever faster. The sequence of the first bacterial genome (Fleischmann *et al.*, 1995 ▸) was soon followed by other genome sequences, including the human genome sequence (Lander *et al.*, 2001 ▸). Structural biology could not keep pace with gene sequencing; however, the seemingly achievable concept of structural genomics emerged (Shapiro & Lima, 1998 ▸). Here, the aspiration was for structure determination on a pan-genomic scale, aiming to produce experimental structures representative of each sequence family in all living organisms. These representative structures would serve as templates for homology modeling of particular family members, as needed. MAD and SAD phasing of selenomethionyl proteins became a foundation for efficiency in structural genomics ventures worldwide.

Selenomethionyl proteins were at the heart of the structure-determination pipelines for all four large-scale centers of the NIH Protein Structure Initiative (PSI) [the Joint Center for Structural Genomics (JCSG; van den Bedem *et al.*, 2011 ▸), the Midwest Center for Structural Genomics (MCSG; Kim *et al.*, 2011 ▸), the Northeast Structural Genomics Consortium (NESG; Xiao *et al.*, 2010 ▸) and the New York SGX Research Center for Structural Genomics (NYSGXRC; Bonanno *et al.*, 2005 ▸)] and for the New York Consortium on Membrane Protein Structure (NYCOMPS) membrane-protein center (Love *et al.*, 2010 ▸). Over the ten-year period of the PSI protein-universe effort, PSI-1 and PSI-2 deposited 5097 atomic structures in the Protein Data Bank, of which 4598 (90%) were crystal structures. These crystal structures included 1353 (29%) determined by MAD and 2168 (47%) determined by SAD (Hendrickson, 2014 ▸).

Contributions from structural genomics have had a large impact on the growth of Protein Data Bank (PDB) holdings (Chandonia & Brenner, 2006 ▸; Levitt, 2007 ▸), with MAD and SAD predominating at 69% (Hendrickson, 2014 ▸). Because of the emphasis of structural genomics on sequence families, without regard to biological activity or other properties, structural genomics has had an exceptional impact in structural novelty. Levitt (2007 ▸) estimated that 50% of non­redundant sequence data in the PDB from 2005 until mid-2007 came from structural genomics efforts. In turn, this novelty from MAD and SAD structures was crucial in enabling the remarkable advances in protein structure prediction made by *AlphaFold* (Jumper *et al.*, 2021 ▸).

## 18. Changing practice in macromolecular phasing

At the outset of protein crystallography, all structures were determined by MIR phasing, although anomalous scattering soon entered by way of MIRAS and SIRAS. Many early structures led to isomorphic variants (IVs), including ligand complexes as well as the heavy-atom derivatives used in MIR, and ultimately these included mutant variants and other modifications. Molecular replacement (MR), as we know it now, first appeared with Tollin’s test (Tollin, 1969 ▸) and it was not until the structure of crambin (Hendrickson & Teeter, 1981 ▸) that anomalous scattering was used alone in what we now call SAD. As recounted above, MAD followed in 1985. It was not until 1996 that the PDB began to record depositor declarations on the method of structure determination; however, during the decade 1990–1999 we produced the annual *Macromolecular Structures* (*MS*) series (Hendrickson & Wüthrich, 2000 ▸), in which we published curated abstracts of reported structures meeting the criteria of being a biological macromolecule, novel and experimentally determined at an atomic level. From the *MS* series before 1996 and from PDB statistics after, I was able to chronicle the evolution of crystallographic structure determination (Hendrickson, 2014 ▸).

The record of this progression documents a succession of sea changes in the prevalent usages of phasing methods. MR had not yet appeared when Blundell & Johnson (1976 ▸) ‘wrote the book’ on protein crystallography, but already in *MS*1991 (Hendrickson & Wüthrich, 1991 ▸) we recorded that 51% of *MS*-qualifying structures had been determined by MR, MIR dominated for *de novo* phasing and only a few MAD structures had been reported. At the expense of MIR, MAD had risen to 32% of *de novo* structures by 1999 (*MS*2000), and this rise continued as deduced from PDB statistics [Fig. 14 ▸(*a*)]. MAD overtook the combination of MIR and SIR in 2000, and SAD overtook MAD in 2006. Whereas the isomorphous-replacement share (MIR + SIR) was over 80% at the start of recordings in 1997, this predominance had reversed to ∼90% for anomalous diffraction (MAD + SAD) from 2008 onwards. By 2013, SAD alone accounted for ∼70% of all *de novo* structures. I have estimated that 77% of all *de novo* crystal structures of macromolecules accumulated through 2012 were determined by MAD or SAD phasing (Hendrickson, 2014 ▸).

**Figure 15:**
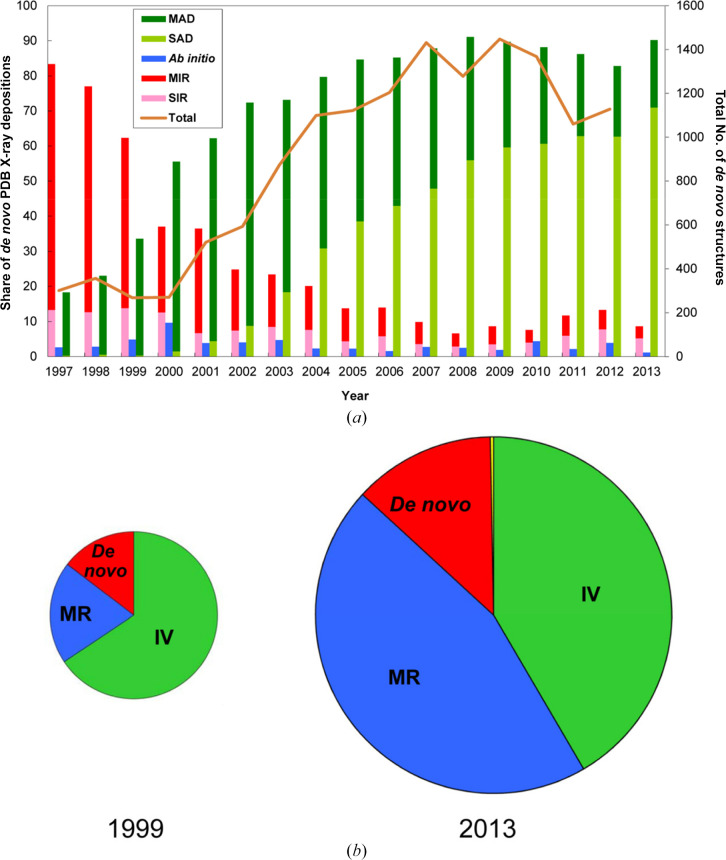
Changing practice in macromolecular structure determination. (*a*) *De novo* PDB depositions. Fractional contributions are shown, year by year, from isomorphous replacement, MIR (red) and SIR (pink), compared with anomalous diffraction, MAD (green) and SAD (chartreuse), and with *ab initio* methods (blue). We parsed depositor declarations to the PDB from 1997 through 10 December 2013, counting multiple declarations into each. The orange line traces the total number of *de novo* depositions with time. (*b*) Pie charts of the major categories of PDB depositions (*de novo*, MR for molecular replacement and IV for isomorphous variant) in 1999 and in 2013. The area of each pie is proportional to the total number of depositions in that year. The division between MR and IV is from the *MS*2000 curation for 1999 and was hand curated from 67% declared as MR to 44% being in a novel lattice. (*a*) is reproduced from Hendrickson (2014 ▸) with permission from Cambridge University Press.

Over the period 1999–2013 [Fig. 15 ▸(*b*)] there was substantial growth in PDB depositions; however, the fraction of structures obtained by *de novo* phasing remained essentially constant. What changed dramatically was the portion attributed to MR. In the *MS* series, we distinguished an IV from an MR. An IV is defined as a new structure in a previously described lattice, which should not require new phase evaluation, whereas an MR analysis requires a molecular search and model generation in the new lattice. For some time, many investigators have used MR procedures to solve any new structure, even if in a lattice that is isomorphic with a known structure; often, the resulting structure is then declared to be determined by MR upon PDB deposition. By PDB declaration, MR accounted for 34% of 1999 PDB depositions, compared with 20% by *MS*2000 definition. For 2013, MR was declared for 67% of PDB depositions, whereas by my manual curation the MR fraction was 44%, as plotted in Fig. 15 ▸(*b*).

## 19. Phase evaluation today

Macromolecular crystallography is in a state of comfortable and productive maturity. This, however, is also how it felt in 1971 at the watershed Cold Spring Harbor meeting as the fruits of MIR were on resplendent display (Watson, 1972 ▸). The prospective impact of the now predominating methods of molecular replacement (MR) and anomalous diffraction (MAD and SAD) was nowhere evident. Meanwhile, advances in molecular biology (gene sequences, recombinant proteins, mutational tests of structure-inspired hypotheses and selenomethionyl proteins), instrumentation (computers, molecular graphics, detectors, synchrotron beamlines and FELs) and diffraction methods (restrained refinement, MR, DM, MAD and SAD) effected revolutions in how we practice macromolecular crystallography today. It would be foolhardy to feel comfortable in our current maturity.

Developments in kindred disciplines continue to impact crystallography, and crystallography reciprocates. Recent advances in cryogenic electron microscopy (cryo-EM) and protein structure prediction (*AlphaFold*) relate cogently to crystallographic phase evaluation.

The ‘resolution revolution’ in cryo-EM (Kühlbrandt, 2014 ▸) quickly spawned remarkable advances in structural biology (Saibil, 2022 ▸). Direct imaging, even at a modest 4 Å resolution, was awe-inspiring to those who have faced the phase problem in crystallographic imaging. Most macromolecular crystallographers have become electron microscopists as well; this is much to the benefit of advancing cryo-EM, and the cryo-EM images are inspirational for advancing crystallo­graphy. X-ray crystallography can complement cryo-EM. A noteworthy example comes in element identification, notably for bound ions. Cryo-EM is problematic in this regard, whereas refinement of the *f*′′ anomalous scattering contribution from a site is usually definitive (Liu *et al.*, 2013 ▸; Vecchioni *et al.*, 2023 ▸).

I made the case above that MAD- and SAD-phased structures have contributed 77% of the novel PDB content (evaluated through 2012) on which the neural-network training needed to perfect *AlphaFold* was based (Jumper *et al.*, 2021 ▸). The effectiveness of *AlphaFold* is widely appreciated in modern biology generally, and notably so for macromolecular crystallography. *AlphaFold* models immediately became the search models of choice for molecular replacement (McCoy *et al.*, 2022 ▸; Gong *et al.*, 2023 ▸; Terwilliger *et al.*, 2023 ▸). *AlphaFold* accuracy is such that this development is expected to expand the reach of MR.

MAD and SAD will be at the ready for otherwise unmet challenges; however, cryo-EM, *AlphaFold* and future advances will likely decrease the dependence on crystallo­graphic phase evaluation. Just as MIR gave way to MAD, and MAD gave way to SAD (even native SAD), SAD may give way to *AlphaFold*-guided MR.

## References

[bb1] Abbe, E. (1873). *Arch. Mikrosk. Anat.* **9**, 413–468.

[bb2] Abrahams, J. P. & Leslie, A. G. W. (1996). *Acta Cryst.* D**52**, 30–42. 10.1107/S0907444995008754 15299723

[bb3] Akey, D. L., Terwilliger, T. C. & Smith, J. L. (2016). *Acta Cryst.* D**72**, 296–302. 10.1107/S2059798315021920 PMC4784661 26960117

[bb4] Als-Nielsen, J. & McMorrow, D. (2011). *Elements of Modern X-ray Physics*, 2nd ed. Chichester: John Wiley & Sons.

[bb5] Assur Sanghai, Z., Liu, Q., Clarke, O. B., Belcher-Dufrisne, M., Wiriyasermkul, P., Giese, M. H., Leal-Pinto, E., Kloss, B., Tabuso, S., Love, J., Punta, M., Banerjee, S., Rajashankar, K. R., Rost, B., Logothetis, D., Quick, M., Hendrickson, W. A. & Mancia, F. (2018). *eLife*, **7**, e27829. 10.7554/eLife.27829 PMC5967866 29792261

[bb6] Astbury, W. T. & Street, A. (1931). *Philos. Trans. R. Soc. London A*, **230**, 75–101.

[bb7] Athappilly, F. K. & Hendrickson, W. A. (1995). *Structure*, **3**, 1407–1419. 10.1016/s0969-2126(01)00277-5 8747466

[bb8] Aurelius, O., Duman, R., El Omari, K., Mykhaylyk, V. & Wagner, A. (2017). *Nucl. Instrum. Methods Phys. Res. B*, **411**, 12–16. 10.1016/j.nimb.2016.12.005 PMC5727679 29276323

[bb9] Basu, S., Olieric, V., Leonarski, F., Matsugaki, N., Kawano, Y., Takashi, T., Huang, C.-Y., Yamada, Y., Vera, L., Olieric, N., Basquin, J., Wojdyla, J. A., Bunk, O., Diederichs, K., Yamamoto, M. & Wang, M. (2019). *IUCrJ*, **6**, 373–386. 10.1107/S2052252519002756 PMC6503925 31098019

[bb10] Bedem, H. van den, Wolf, G., Xu, Q. & Deacon, A. M. (2011). *Acta Cryst.* D**67**, 368–375. 10.1107/S0907444910039934 PMC3069752 21460455

[bb11] Beevers, C. A. & Lipson, H. (1934). *London Edinb. Dubl. Philos. Mag. J. Sci.* **17**, 855–859.

[bb12] Beevers, C. A. & Lipson, H. (1935). *Proc. R. Soc. London A*, **148**, 664–680.

[bb13] Bernal, J. D. & Crowfoot, D. (1934). *Nature*, **133**, 794–795.

[bb14] Bijvoet, J. M. (1949). *Proc. Acad. Sci. Amst. B*, **52**, 313–314.

[bb15] Bijvoet, J. M. (1954). *Nature*, **173**, 888–891.

[bb16] Bijvoet, J. M., Peerdeman, A. F. & van Bommel, A. J. (1951). *Nature*, **168**, 271–272.

[bb17] Biou, V., Shu, F. & Ramakrishnan, V. (1995). *EMBO J.* **14**, 4056–4064. 10.1002/j.1460-2075.1995.tb00077.x PMC394484 7664745

[bb18] Blow, D. M. (1958). *Proc. R. Soc. London A*, **247**, 302–336.

[bb19] Blow, D. M. & Crick, F. H. C. (1959). *Acta Cryst.* **12**, 794–802.

[bb20] Blow, D. M. & Rossmann, M. G. (1961). *Acta Cryst.* **14**, 1195–1202.

[bb21] Blundell, T. L. & Johnson, L. N. (1976). *Protein Crystallography*. New York: Academic Press.

[bb22] Bodo, G., Dintzis, H. M., Kendrew, J. C. & Wyckoff, H. W. (1959). *Proc. R. Soc. London A*, **253**, 70–102.

[bb23] Bohr, N. (1913). *London Edinb. Dubl. Philos. Mag. J. Sci.* **26**, 1–25.

[bb24] Bokhoven, C., Schoone, J. C. & Bijvoet, J. M. (1951). *Acta Cryst.* **4**, 275–280.

[bb25] Bonanno, J. B., Almo, S. C., Bresnick, A., Chance, M. R., Fiser, A., Swaminathan, S., Jiang, J., Studier, F. W., Shapiro, L., Lima, C. D., Gaasterland, T. M., Sali, A., Bain, K., Feil, I., Gao, X., Lorimer, D., Ramos, A., Sauder, J. M., Wasserman, S. R., Emtage, S., D’Amico, K. L. & Burley, S. K. (2005). *J. Struct. Funct. Genomics*, **6**, 225–232. 10.1007/s10969-005-6827-0 16211523

[bb26] Bragg, W. H. (1915). *Philos. Trans. R. Soc. London A*, **215**, 253–274.

[bb27] Bragg, W. H. & Bragg, W. L. (1913). *Proc. R. Soc. London A*, **89**, 277–291.

[bb28] Bragg, W. L. (1913*a*). *Proc. Camb. Philos. Soc.* **17**, 43–57.

[bb29] Bragg, W. L. (1913*b*). *Proc. R. Soc. London A*, **89**, 246–248.

[bb30] Bragg, W. L. (1914). *Proc. R. Soc. London A*, **89**, 468–489.

[bb31] Bragg, W. L. (1929). *Proc. R. Soc. London A*, **123**, 537–559.

[bb880] Bricogne, G. (1976). *Acta Cryst.* A**32**, 832–847.

[bb32] Brodersen, D. E., de La Fortelle, E., Vonrhein, C., Bricogne, G., Nyborg, J. & Kjeldgaard, M. (2000). *Acta Cryst.* D**56**, 431–441. 10.1107/s0907444900000834 10739916

[bb33] Brünger, A. T. (1991). *Annu. Rev. Phys. Chem.* **42**, 197–223.

[bb34] Carlisle, C. H. & Crowfoot, D. (1945). *Proc. R. Soc. London A*, **184**, 64–83.

[bb881] Champness, J. N., Bloomer, A. C., Bricogne, G., Butler, P. J. G. & Klug, A. (1976). *Nature*, **259**, 20–24. 10.1038/259020a0 1250335

[bb35] Chandonia, J. M. & Brenner, S. E. (2006). *Science*, **311**, 347–351. 10.1126/science.1121018 16424331

[bb36] Chang, Y., Bruni, R., Kloss, B., Assur, Z., Kloppmann, E., Rost, B., Hendrickson, W. A. & Liu, Q. (2014). *Science*, **344**, 1131–1135. 10.1126/science.1252043 PMC4119810 24904158

[bb37] Chen, L., Rose, J. P., Breslow, E., Yang, D., Chang, W. R., Furey, W. F. Jr, Sax, M. & Wang, B.-C. (1991). *Proc. Natl Acad. Sci. USA*, **88**, 4240–4244. 10.1073/pnas.88.10.4240 PMC51634 2034668

[bb38] Chen, Y. H., Hu, L., Punta, M., Bruni, R., Hillerich, B., Kloss, B., Rost, B., Love, J., Siegelbaum, S. A. & Hendrickson, W. A. (2010). *Nature*, **467**, 1074–1080. 10.1038/nature09487 PMC3548404 20981093

[bb39] Cheung, J., Bingman, C. A., Reyngold, M., Hendrickson, W. A. & Waldburger, C. D. (2008). *J. Biol. Chem.* **283**, 13762–13770. 10.1074/jbc.M710592200 PMC2376233 18348979

[bb40] Cheung, J. & Hendrickson, W. A. (2008). *J. Biol. Chem.* **283**, 30256–30265. 10.1074/jbc.M805253200 PMC2573060 18701447

[bb41] Cheung, J. & Hendrickson, W. A. (2009). *Structure*, **17**, 190–201. 10.1016/j.str.2008.12.013 PMC3749045 19217390

[bb42] Cheung, J., Le-Khac, M. & Hendrickson, W. A. (2009). *Proteins*, **77**, 235–241. 10.1002/prot.22485 PMC2730989 19544572

[bb43] Collins, M. N. & Hendrickson, W. A. (2011). *Structure*, **19**, 324–336. 10.1016/j.str.2010.11.017 PMC3099344 21397184

[bb44] Coster, D., Knol, K. S. & Prins, J. A. (1930). *Z. Phys.* **63**, 345–369.

[bb45] Cowie, D. B. & Cohen, G. N. (1957). *Biochim. Biophys. Acta*, **26**, 252–261. 10.1016/0006-3002(57)90003-3 13499359

[bb46] Cowtan, K. (2010). *Acta Cryst.* D**66**, 470–478. 10.1107/S090744490903947X PMC2852311 20383000

[bb47] Cowtan, K. D. & Main, P. (1993). *Acta Cryst.* D**49**, 148–157. 10.1107/S0907444992007698 15299555

[bb48] Cromer, D. T. & Liberman, D. (1970). *J. Chem. Phys.* **53**, 1891–1898.

[bb49] Crowfoot, D., Bunn, C. W., Rogers-Low, B. W. & Turner, A. (1949). *Chemistry of Penicillin*, edited by H. T. Clarke, pp. 310–366. Oxford University Press.

[bb301] Crowther, R. A. (1967). *Acta Cryst.* **22**, 758–764.

[bb302] Crowther, R. A. (1972). *The Molecular Replacement Method*, edited by M. G. Rossmann, pp. 173–178. New York: Gordon & Breach.

[bb303] Crowther, R. A. & Blow, D. M. (1967). *Acta Cryst.* **23**, 544–548.

[bb50] Cuff, M. E., Miller, K. I., van Holde, K. E. & Hendrickson, W. A. (1998). *J. Mol. Biol.* **278**, 855–870. 10.1006/jmbi.1998.1647 9614947

[bb51] Dauter, Z. & Adamiak, D. A. (2001). *Acta Cryst.* D**57**, 990–995. 10.1107/s0907444901006382 11418767

[bb52] Dauter, Z. & Dauter, M. (1999). *J. Mol. Biol.* **289**, 93–101. 10.1006/jmbi.1999.2744 10339408

[bb53] Dauter, Z., Dauter, M., de La Fortelle, E., Bricogne, G. & Sheldrick, G. M. (1999). *J. Mol. Biol.* **289**, 83–92. 10.1006/jmbi.1999.2743 10339407

[bb54] Dauter, Z., Dauter, M. & Dodson, E. J. (2002). *Acta Cryst.* D**58**, 494–506. 10.1107/s090744490200118x 11856836

[bb55] Deacon, A. M. & Ealick, S. E. (1999). *Structure*, **7**, R161–R166. 10.1016/s0969-2126(99)80096-3 10425674

[bb56] Deacon, A. M., Ni, Y. S., Coleman, W. G. Jr & Ealick, S. E. (2000). *Structure*, **8**, 453–462. 10.1016/s0969-2126(00)00128-3 10896473

[bb57] DiGabriele, A. D., Lax, I., Chen, D. I., Svahn, C. M., Jaye, M., Schlessinger, J. & Hendrickson, W. A. (1998). *Nature*, **393**, 812–817. 10.1038/31741 9655399

[bb58] Duane, W. (1925). *Proc. Natl Acad. Sci. USA*, **11**, 489–493. 10.1073/pnas.11.8.489 PMC1086084 16576900

[bb59] El Omari, K., Iourin, O., Kadlec, J., Fearn, R., Hall, D. R., Harlos, K., Grimes, J. M. & Stuart, D. I. (2014). *Acta Cryst.* D**70**, 2197–2203. 10.1107/S139900471401339X PMC4118829 25084338

[bb60] Evans, G. & Pettifer, R. F. (2001). *J. Appl. Cryst.* **34**, 82–86.

[bb61] Ewald, P. P. (1913). *Phys. Z.* **14**, 465–472.

[bb62] Ewald, P. P. (1921). *Z. Kristallogr.* **56**, 129–156.

[bb63] Ewald, P. P. & Hermann, C. (1927). *Z. Kristallogr.* **65**, 251–260.

[bb64] Fanchon, E. & Hendrickson, W. A. (1990). *Acta Cryst.* A**46**, 809–820. 10.1107/s0108767390005967 2248730

[bb65] Fleischmann, R. D., Adams, M. D., White, O., Clayton, R. A., Kirkness, E. F., Kerlavage, A. R., Bult, C. J., Tomb, J., Dougherty, B. A., Merrick, J. M., McKenney, K., Sutton, G., FitzHugh, W., Fields, C., Gocayne, J. D., Scott, J., Shirley, R., Liu, L., Glodek, A., Kelley, J. M., Weidman, J. F., Phillips, C. A., Spriggs, T., Hedblom, E., Cotton, M. D., Utterback, T. R., Hanna, M. C., Nguyen, D. T., Saudek, D. M., Brandon, R. C., Fine, L. D., Fritchman, J. L., Fuhrmann, J. L., Geoghagen, N. S. M., Gnehm, C. L., McDonald, L. A., Small, K. V., Fraser, C. M., Smith, H. O. & Venter, J. C. (1995). *Science*, **269**, 496–512.

[bb66] Franklin, R. E. & Gosling, R. G. (1953). *Nature*, **171**, 740–741. 10.1038/171740a0 13054694

[bb67] Fridrichsons, J. & Mathieson, A. M. (1967). *Acta Cryst.* **23**, 439–448. 10.1107/s0365110x67002944 5630459

[bb68] Friedrich, W., Knipping, P. & Laue, M. (1912). *Sitzungber. Bayer. Akad. Wiss.*, pp. 303–322.

[bb69] Georgiadis, M. M., Jessen, S. M., Ogata, C. M., Telesnitsky, A., Goff, S. P. & Hendrickson, W. A. (1995). *Structure*, **3**, 879–892. 10.1016/S0969-2126(01)00223-4 8535782

[bb70] Glaeser, R. M., Downing, K., DeRosier, D., Chiu, W. & Frank, J. (2007). *Electron Crystallography of Biological Macromolecules*, ch. 3. Oxford University Press.

[bb71] Gong, Z., Wang, W., El Omari, K., Lebedev, A. A., Clarke, O. B. & Hendrickson, W. A. (2023). *Proc. Natl Acad. Sci. USA*, **120**, e2218630120. 10.1073/pnas.2218630120 PMC9910494 36574673

[bb72] Green, D. W., Ingram, V. M. & Perutz, M. F. (1954). *Proc. R. Soc. London A*, **225**, 287–307.

[bb73] Guo, Y., Kalathur, R. C., Liu, Q., Kloss, B., Bruni, R., Ginter, C., Kloppmann, E., Rost, B. & Hendrickson, W. A. (2015). *Science*, **347**, 551–555. 10.1126/science.aaa1534 PMC4341906 25635100

[bb74] Guss, J. M., Merritt, E. A., Phizackerley, R. P., Hedman, B., Murata, M., Hodgson, K. & Freeman, H. C. (1988). *Science*, **241**, 806–811. 10.1126/science.3406739 3406739

[bb75] Hall, S. R. & Maslen, E. N. (1965). *Acta Cryst.* **18**, 265–279.

[bb76] Harker, D. (1936). *J. Chem. Phys.* **4**, 381–390.

[bb77] Harker, D. (1956). *Acta Cryst.* **9**, 1–9.

[bb78] Hauptman, H. & Karle, J. (1953). *Solution of the Phase Problem I. The Centrosymmetric Crystal.* Dayton: Polycrystal Book Service.

[bb79] Hendrickson, W. A. (1971). *Acta Cryst.* B**27**, 1474–1475.

[bb80] Hendrickson, W. A. (1979). *Acta Cryst.* A**35**, 245–247.

[bb81] Hendrickson, W. A. (1981). *Structural Aspects of Biomolecules*, edited by R. Srinivasan & V. Pattabhi, pp. 31–80. New Delhi: Macmillan.

[bb82] Hendrickson, W. A. (1984). *Acta Cryst.* A**40**, C3.

[bb83] Hendrickson, W. A. (1985). *Trans. Am. Crystallogr. Assoc.* **21**, 11–21.

[bb84] Hendrickson, W. A. (1991). *Science*, **254**, 51–58. 10.1126/science.1925561 1925561

[bb85] Hendrickson, W. A. (2014). *Q. Rev. Biophys.* **47**, 49–93. 10.1017/S0033583514000018 PMC4128195 24726017

[bb86] Hendrickson, W. A., Co, M. S., Smith, J. L., Hodgson, K. O. & Klippenstein, G. L. (1982). *Proc. Natl Acad. Sci. USA*, **79**, 6255–6259. 10.1073/pnas.79.20.6255 PMC347099 6959115

[bb87] Hendrickson, W. A., Horton, J. R. & LeMaster, D. M. (1990). *EMBO J.* **9**, 1665–1672. 10.1002/j.1460-2075.1990.tb08287.x PMC551863 2184035

[bb88] Hendrickson, W. A. & Karle, J. (1973). *J. Biol. Chem.* **248**, 3327–3334. 4735582

[bb89] Hendrickson, W. A., Klippenstein, G. L. & Ward, K. B. (1975). *Proc. Natl Acad. Sci. USA*, **72**, 2160–2164. 10.1073/pnas.72.6.2160 PMC432716 1056020

[bb90] Hendrickson, W. A. & Konnert, J. H. (1980). *Computing in Crystallography*, edited by R. Diamond, S. Rameseshan & K. Venkatesan, pp. 13.01–13.23. Bangalore: Indian Academy of Sciences.

[bb91] Hendrickson, W. A. & Lattman, E. E. (1970). *Acta Cryst.* B**26**, 136–143.

[bb92] Hendrickson, W. A. & Ogata, C. M. (1997). *Methods Enzymol.* **276**, 494–523. 10.1016/S0076-6879(97)76074-9 27799111

[bb93] Hendrickson, W. A., Pähler, A., Smith, J. L., Satow, Y., Merritt, E. A. & Phizackerley, R. P. (1989). *Proc. Natl Acad. Sci. USA*, **86**, 2190–2194. 10.1073/pnas.86.7.2190 PMC286877 2928324

[bb94] Hendrickson, W. A., Smith, J. L., Phizackerley, R. P. & Merritt, E. A. (1988). *Proteins*, **4**, 77–88. 10.1002/prot.340040202 3227016

[bb95] Hendrickson, W. A. & Teeter, M. M. (1981). *Nature*, **290**, 107–113. 10.1038/290107a0 PMC5536114 28769131

[bb305] Hendrickson, W. A. & Ward, K. B. (1976). *Acta Cryst.* A**32**, 778–780.

[bb304] Hendrickson, W. A. & Ward, K. B. (1977). *J. Biol. Chem.* **252**, 3012–3018. 856811

[bb96] Hendrickson, W. A. & Wüthrich, K. (1991). Editors. *Macromolecular Structures 1991: Atomic Structures of Biological Macromolecules Reported During 1990.* London: Current Biology.

[bb97] Hendrickson, W. A. & Wüthrich, K. (2000). Editors. *Macromolecular Structures 2000: Atomic Structures of Biological Macromolecules Reported During 1999.* London: Current Biology.

[bb98] Herriott, J. R., Sieker, L. C., Jensen, L. H. & Lovenberg, W. (1970). *J. Mol. Biol.* **50**, 391–406. 10.1016/0022-2836(70)90200-7 5476919

[bb99] Hodgkin, D. C., Kamper, J., Lindsey, J., MacKay, M., Pickworth, J., Robertson, J. H., Shoemaker, C. B., White, J. G., Prosen, R. J. & Trueblood, K. N. (1957). *Proc. R. Soc. London A*, **242**, 228–263.

[bb100] Hönl, H. (1933). *Z. Phys.* **84**, 1–16.

[bb101] Hope, H. (1988). *Acta Cryst.* B**44**, 22–26. 10.1107/s0108768187008632 3271102

[bb102] Hoppe, W. & Jakubowski, V. (1971). *Anomalous Scattering*, edited by S. Ramaseshan & S. Abrahams, pp. 437–461. Copenhagen: Munksgaard.

[bb103] Hubbard, S. R., Wei, L., Ellis, L. & Hendrickson, W. A. (1994). *Nature*, **372**, 746–754. 10.1038/372746a0 7997262

[bb104] Jack, A. & Levitt, M. (1978). *Acta Cryst.* A**34**, 931–935.

[bb105] Jiang, X., Gurel, O., Mendiaz, E. A., Stearns, G. W., Clogston, C. L., Lu, H. S., Osslund, T. D., Syed, R. S., Langley, K. E. & Hendrickson, W. A. (2000). *EMBO J.* **19**, 3192–3203. 10.1093/emboj/19.13.3192 PMC313947 10880433

[bb106] Jumper, J., Evans, R., Pritzel, A., Green, T., Figurnov, M., Ronneberger, O., Tunyasuvunakool, K., Bates, R., Žídek, A., Potapenko, A., Bridgland, A., Meyer, C., Kohl, S. A. A., Ballard, A. J., Cowie, A., Romera-Paredes, B., Nikolov, S., Jain, R., Adler, J., Back, T., Petersen, S., Reiman, D., Clancy, E., Zielinski, M., Steinegger, M., Pacholska, M., Berghammer, T., Bodenstein, S., Silver, D., Vinyals, O., Senior, A. W., Kavukcuoglu, K., Kohli, P. & Hassabis, D. (2021). *Nature*, **596**, 583–589. 10.1038/s41586-021-03819-2 PMC8371605 34265844

[bb107] Kahn, R., Fourme, R., Bosshard, R., Chiadmi, M., Risler, J. L., Dideberg, O. & Wery, J. P. (1985). *FEBS Lett.* **179**, 133–137. 10.1016/0014-5793(85)80207-6 3965297

[bb108] Karasawa, A., Andi, B., Fuchs, M. R., Shi, W., McSweeney, S., Hendrickson, W. A. & Liu, Q. (2022). *IUCrJ*, **9**, 768–777. 10.1107/S205225252200971X PMC9634608 36381147

[bb109] Karle, J. (1967). *Appl. Opt.* **6**, 2132–2135. 10.1364/AO.6.002132 20062375

[bb110] Karle, J. & Hauptman, H. (1950). *Acta Cryst.* **3**, 181–187.

[bb111] Karle, J. & Hauptman, H. (1956). *Acta Cryst.* **9**, 635–651.

[bb112] Kendrew, J. C., Bodo, G., Dintzis, H. M., Parrish, R. G., Wyckoff, H. & Phillips, D. C. (1958). *Nature*, **181**, 662–666. 10.1038/181662a0 13517261

[bb113] Kim, M. S., Lapkouski, M., Yang, W. & Gellert, M. (2015). *Nature*, **518**, 507–511. 10.1038/nature14174 PMC4342785 25707801

[bb114] Kim, Y., Babnigg, G., Jedrzejczak, R., Eschenfeldt, W. H., Li, H., Maltseva, N., Hatzos-Skintges, C., Gu, M., Makowska-Grzyska, M., Wu, R., An, H., Chhor, G. & Joachimiak, A. (2011). *Methods*, **55**, 12–28. 10.1016/j.ymeth.2011.07.010 PMC3690762 21907284

[bb115] Konnert, J. H. & Hendrickson, W. A. (1980). *Acta Cryst.* A**36**, 344–350.

[bb116] Kovall, R. A. & Hendrickson, W. A. (2004). *EMBO J.* **23**, 3441–3451. 10.1038/sj.emboj.7600349 PMC516623 15297877

[bb117] Kramers, H. A. (1924). *Nature*, **113**, 673–674.

[bb118] Kronig, R. I. & Kramers, H. A. (1928). *Z. Phys.* **48**, 174–179.

[bb119] Kühlbrandt, W. (2014). *Science*, **343**, 1443–1444. 10.1126/science.1251652 24675944

[bb120] Lander, E. S., Linton, L. M., Birren, B., Nusbaum, C., Zody, M. C., Baldwin, J., Devon, K., Dewar, K., Doyle, M., FitzHugh, W., Funke, R., Gage, D., Harris, K., Heaford, A., Howland, J., Kann, L., Lehoczky, J., LeVine, R., McEwan, P., McKernan, K., Meldrim, J., Mesirov, J. P., Miranda, C., Morris, W., Naylor, J., Raymond, C., Rosetti, M., Santos, R., Sheridan, A., Sougnez, C., Stange-Thomann, Y., Stojanovic, N., Subramanian, A., Wyman, D., Rogers, J., Sulston, J., Ainscough, R., Beck, S., Bentley, D., Burton, J., Clee, C., Carter, N., Coulson, A., Deadman, R., Deloukas, P., Dunham, A., Dunham, I., Durbin, R., French, L., Grafham, D., Gregory, S., Hubbard, T., Humphray, S., Hunt, A., Jones, M., Lloyd, C., McMurray, A., Matthews, L., Mercer, S., Milne, S., Mullikin, J. C., Mungall, A., Plumb, R., Ross, M., Shownkeen, R., Sims, S., Waterston, R. H., Wilson, R. K., Hillier, L. W., McPherson, J. D., Marra, M. A., Mardis, E. R., Fulton, L. A., Chinwalla, A. T., Pepin, K. H., Gish, W. R., Chissoe, S. L., Wendl, M. C., Delehaunty, K. D., Miner, T. L., Delehaunty, A., Kramer, J. B., Cook, L. L., Fulton, R. S., Johnson, D. L., Minx, P. J., Clifton, S. W., Hawkins, T., Branscomb, E., Predki, P., Richardson, P., Wenning, S., Slezak, T., Doggett, N., Cheng, J. F., Olsen, A., Lucas, S., Elkin, C., Uberbacher, E., Frazier, M., Gibbs, R. A., Muzny, D. M., Scherer, S. E., Bouck, J. B., Sodergren, E. J., Worley, K. C., Rives, C. M., Gorrell, J. H., Metzker, M. L., Naylor, S. L., Kucherlapati, R. S., Nelson, D. L., Weinstock, G. M., Sakaki, Y., Fujiyama, A., Hattori, M., Yada, T., Toyoda, A., Itoh, T., Kawagoe, C., Watanabe, H., Totoki, Y., Taylor, T., Weissenbach, J., Heilig, R., Saurin, W., Artiguenave, F., Brottier, P., Bruls, T., Pelletier, E., Robert, C., Wincker, P., Smith, D. R., Doucette-Stamm, L., Rubenfield, M., Weinstock, K., Lee, H. M., Dubois, J., Rosenthal, A., Platzer, M., Nyakatura, G., Taudien, S., Rump, A., Yang, H., Yu, J., Wang, J., Huang, G., Gu, J., Hood, L., Rowen, L., Madan, A., Qin, S., Davis, R. W., Federspiel, N. A., Abola, A. P., Proctor, M. J., Myers, R. M., Schmutz, J., Dickson, M., Grimwood, J., Cox, D. R., Olson, M. V., Kaul, R., Raymond, C., Shimizu, N., Kawasaki, K., Minoshima, S., Evans, G. A., Athanasiou, M., Schultz, R., Roe, B. A., Chen, F., Pan, H., Ramser, J., Lehrach, H., Reinhardt, R., McCombie, W. R., de la Bastide, M., Dedhia, N., Blöcker, H., Hornischer, K., Nordsiek, G., Agarwala, R., Aravind, L., Bailey, J. A., Bateman, A., Batzoglou, S., Birney, E., Bork, P., Brown, D. G., Burge, C. B., Cerutti, L., Chen, H. C., Church, D., Clamp, M., Copley, R. R., Doerks, T., Eddy, S. R., Eichler, E. E., Furey, T. S., Galagan, J., Gilbert, J. G., Harmon, C., Hayashizaki, Y., Haussler, D., Hermjakob, H., Hokamp, K., Jang, W., Johnson, L. S., Jones, T. A., Kasif, S., Kaspryzk, A., Kennedy, S., Kent, W. J., Kitts, P., Koonin, E. V., Korf, I., Kulp, D., Lancet, D., Lowe, T. M., McLysaght, A., Mikkelsen, T., Moran, J. V., Mulder, N., Pollara, V. J., Ponting, C. P., Schuler, G., Schultz, J., Slater, G., Smit, A. F., Stupka, E., Szustakowki, J., Thierry-Mieg, D., Thierry-Mieg, J., Wagner, L., Wallis, J., Wheeler, R., Williams, A., Wolf, Y. I., Wolfe, K. H., Yang, S. P., Yeh, R. F., Collins, F., Guyer, M. S., Peterson, J., Felsenfeld, A., Wetterstrand, K. A., Patrinos, A., Morgan, M. J., de Jong, P., Catanese, J. J., Osoegawa, K., Shizuya, H., Choi, S., Chen, Y. J., Szustakowki, J. & International Human Genome Sequencing Consortium (2001). *Nature*, **409**, 860–921.

[bb121] Lattman, E. E., Nockolds, C. E., Kretsinger, R. H. & Love, W. E. (1971). *J. Mol. Biol.* **60**, 271–277. 10.1016/0022-2836(71)90293-2 5107328

[bb122] Leahy, D. J., Hendrickson, W. A., Aukhil, I. & Erickson, H. P. (1992). *Science*, **258**, 987–991. 10.1126/science.1279805 1279805

[bb123] Levitt, M. (2007). *Proc. Natl Acad. Sci. USA*, **104**, 3183–3188. 10.1073/pnas.0611678104 PMC1802002 17360626

[bb124] Lima, C. D., D’Amico, K. L., Naday, I., Rosenbaum, G., Westbrook, E. M. & Hendrickson, W. A. (1997). *Structure*, **5**, 763–774. 10.1016/s0969-2126(97)00231-1 9261067

[bb126] Liu, Q., Dahmane, T., Zhang, Z., Assur, Z., Brasch, J., Shapiro, L., Mancia, F. & Hendrickson, W. A. (2012). *Science*, **336**, 1033–1037. 10.1126/science.1218753 PMC3769101 22628655

[bb128] Liu, Q. & Hendrickson, W. A. (2007). *Cell*, **131**, 106–120. 10.1016/j.cell.2007.08.039 PMC2041797 17923091

[bb127] Liu, Q. & Hendrickson, W. A. (2015). *Curr. Opin. Struct. Biol.* **34**, 99–107. 10.1016/j.sbi.2015.08.003 PMC4684729 26432413

[bb125] Liu, Q., Liu, Q. & Hendrickson, W. A. (2013). *Acta Cryst.* D**69**, 1314–1332. 10.1107/S0907444913001479 PMC3689535 23793158

[bb129] Liu, Y., Ogata, C. M. & Hendrickson, W. A. (2001). *Proc. Natl Acad. Sci. USA*, **98**, 10648–10653. 10.1073/pnas.191003998 PMC58520 11526210

[bb130] Liu, Z.-J., Vysotski, E. S., Vysotski, E. S., Chen, C.-J., Rose, J. P., Lee, J. & Wang, B.-C. (2000). *Protein Sci.* **9**, 2085–2093. 10.1110/ps.9.11.2085 PMC2144499 11152120

[bb131] Lonsdale, K. (1929). *Proc. R. Soc. London A*, **123**, 494–515.

[bb132] Lonsdale, K. (1931). *Proc. R. Soc. London A*, **133**, 536–552.

[bb133] Love, J., Mancia, F., Shapiro, L., Punta, M., Rost, B., Girvin, M., Wang, D. N., Zhou, M., Hunt, J. F., Szyperski, T., Gouaux, E., MacKinnon, R., McDermott, A., Honig, B., Inouye, M., Montelione, G. & Hendrickson, W. A. (2010). *J. Struct. Funct. Genomics*, **11**, 191–199. 10.1007/s10969-010-9094-7 PMC3099345 20690043

[bb134] Luzzati, V. (1952). *Acta Cryst.* **5**, 802–810.

[bb135] Lye, R. C., Phillips, J. C., Kaplan, D., Doniach, S. & Hodgson, K. O. (1980). *Proc. Natl Acad. Sci. USA*, **77**, 5884–5888. 10.1073/pnas.77.10.5884 PMC350176 6934520

[bb306] Main, P. & Rossmann, M. G. (1966). *Acta Cryst.* **21**, 67–72.

[bb136] Marina, A., Mott, C., Auyzenberg, A., Hendrickson, W. A. & Waldburger, C. D. (2001). *J. Biol. Chem.* **276**, 41182–41190. 10.1074/jbc.M106080200 11493605

[bb137] Marina, A., Waldburger, C. D. & Hendrickson, W. A. (2005). *EMBO J.* **24**, 4247–4259. 10.1038/sj.emboj.7600886 PMC1356327 16319927

[bb138] Mark, H. & Szilard, L. (1925). *Z. Phys.* **33**, 688–691.

[bb139] Martinez-Hackert, E. & Hendrickson, W. A. (2007). *Acta Cryst.* D**63**, 536–547. 10.1107/S090744490700964X 17372359

[bb140] Martinez-Hackert, E. & Hendrickson, W. A. (2009). *Cell*, **138**, 923–934. 10.1016/j.cell.2009.07.044 PMC2799252 19737520

[bb141] Martinez-Hackert, E. & Hendrickson, W. A. (2011). *J. Mol. Biol.* **407**, 450–464. 10.1016/j.jmb.2011.01.027 PMC3099347 21262232

[bb142] Matthews, B. W. (1966). *Acta Cryst.* **20**, 82–86.

[bb307] McCoy, A. J., Grosse-Kunstleve, R. W., Adams, P. D., Winn, M. D., Storoni, L. C. & Read, R. J. (2007). *J. Appl. Cryst.* **40**, 658–674. 10.1107/S0021889807021206 PMC2483472 19461840

[bb143] McCoy, A. J., Sammito, M. D. & Read, R. J. (2022). *Acta Cryst.* D**78**, 1–13. 10.1107/S2059798321012122 PMC8725160 34981757

[bb144] McDonald, N. Q., Panayotatos, N. & Hendrickson, W. A. (1995). *EMBO J.* **14**, 2689–2699. 10.1002/j.1460-2075.1995.tb07269.x PMC398387 7796798

[bb145] Miller, R., Gallo, S. M., Khalak, H. G. & Weeks, C. M. (1994). *J. Appl. Cryst.* **27**, 613–621.

[bb146] Min, K. C., Kovall, R. A. & Hendrickson, W. A. (2003). *Proc. Natl Acad. Sci. USA*, **100**, 14713–14718. 10.1073/pnas.2136684100 PMC299775 14657365

[bb147] Mitchell, C. M. (1957). *Acta Cryst.* **10**, 475–476.

[bb148] Moncrief, J. W. & Lipscomb, W. N. (1966). *Acta Cryst.* **21**, 322–331. 10.1107/s0365110x66002883 5953637

[bb149] Moore, J. O. & Hendrickson, W. A. (2009). *Structure*, **17**, 1195–1204. 10.1016/j.str.2009.07.015 19748340

[bb150] Moore, J. O. & Hendrickson, W. A. (2012). *Structure*, **20**, 729–741. 10.1016/j.str.2012.02.021 PMC3625974 22483119

[bb151] Muirhead, H. & Perutz, M. F. (1963). *Nature*, **199**, 633–638. 10.1038/199633a0 14074546

[bb152] Nass, K., Cheng, R., Vera, L., Mozzanica, A., Redford, S., Ozerov, D., Basu, S., James, D., Knopp, G., Cirelli, C., Martiel, I., Casadei, C., Weinert, T., Nogly, P., Skopintsev, P., Usov, I., Leonarski, F., Geng, T., Rappas, M., Doré, A. S., Cooke, R., Nasrollahi Shirazi, S., Dworkowski, F., Sharpe, M., Olieric, N., Bacellar, C., Bohinc, R., Steinmetz, M. O., Schertler, G., Abela, R., Patthey, L., Schmitt, B., Hennig, M., Standfuss, J., Wang, M. & Milne, C. J. (2020). *IUCrJ*, **7**, 965–975. 10.1107/S2052252520011379 PMC7642782 33209311

[bb153] North, A. C. T. (1965). *Acta Cryst.* **18**, 212–216.

[bb154] Okaya, Y. & Pepinsky, R. (1956). *Phys. Rev.* **103**, 1645–1647.

[bb155] Ormö, M., Cubitt, A. B., Kallio, K., Gross, L. A., Tsien, R. Y. & Remington, S. J. (1996). *Science*, **273**, 1392–1395. 10.1126/science.273.5280.1392 8703075

[bb156] Otwinowski, Z. (1991). *Proceedings of the CCP4 Study Weekend. Isomorphous Replacement and Anomalous Scattering*, edited by W. Wolf, P. R. Evans & A. G. W. Leslie, pp. 80–86. Warrington: Daresbury Laboratory.

[bb157] Pähler, A., Smith, J. L. & Hendrickson, W. A. (1990). *Acta Cryst.* A**46**, 537–540. 10.1107/s0108767390002379 2206480

[bb158] Patterson, A. L. (1934). *Phys. Rev.* **46**, 372–376.

[bb159] Pauling, L. & Corey, R. B. (1951). *Proc. Natl Acad. Sci. USA*, **37**, 251–256. 10.1073/pnas.37.5.251 PMC1063350 14834147

[bb160] Pauling, L., Corey, R. B. & Branson, H. R. (1951). *Proc. Natl Acad. Sci. USA*, **37**, 205–211. 10.1073/pnas.37.4.205 PMC1063337 14816373

[bb161] Peat, T. S., Frank, E. G., McDonald, J. P., Levine, A. S., Woodgate, R. & Hendrickson, W. A. (1996). *Nature*, **380**, 727–730. 10.1038/380727a0 8614470

[bb162] Perrakis, A., Morris, R. & Lamzin, V. S. (1999). *Nat. Struct. Biol.* **6**, 458–463. 10.1038/8263 10331874

[bb163] Phillips, J. C. & Hodgson, K. O. (1980). *Acta Cryst.* A**36**, 856–864.

[bb164] Phillips, J. C., Templeton, D. H., Templeton, L. K. & Hodgson, K. O. (1978). *Science*, **201**, 257–259. 10.1126/science.201.4352.257 17778657

[bb165] Porter, A. B. (1906). *London Edinb. Dubl. Philos. Mag. J. Sci.* **11**, 154–166.

[bb166] Qi, R., Sarbeng, E. B., Liu, Q., Le, K. Q., Xu, X., Xu, H., Yang, J., Wong, J. L., Vorvis, C., Hendrickson, W. A., Zhou, L. & Liu, Q. (2013). *Nat. Struct. Mol. Biol.* **20**, 900–907. 10.1038/nsmb.2583 PMC3772632 23708608

[bb167] Ramachandran, G. N. & Raman, S. (1956). *Curr. Sci.* **25**, 348–351.

[bb168] Ramakrishnan, V. & Biou, V. (1997). *Methods Enzymol.* **276**, 538–557. 9048381

[bb169] Ramakrishnan, V., Finch, J. T., Graziano, V., Lee, P. L. & Sweet, R. M. (1993). *Nature*, **362**, 219–223. 10.1038/362219a0 8384699

[bb170] Raman, S. (1959). *Proc. Indian Acad. Sci. A*, **50**, 95–107.

[bb171] Ramaseshan, S., Venkatesan, K. & Mani, N. V. (1957). *Proc. Indian Acad. Sci.* **46**, 95–111.

[bb172] Read, R. J. (1986). *Acta Cryst.* A**42**, 140–149.

[bb308] Read, R. J. (2001). *Acta Cryst.* D**57**, 1373–1382. 10.1107/s0907444901012471 11567148

[bb173] Read, R. J. & McCoy, A. J. (2011). *Acta Cryst.* D**67**, 338–344. 10.1107/S0907444910051371 PMC3069749 21460452

[bb174] Rice, L. M., Earnest, T. N. & Brunger, A. T. (2000). *Acta Cryst.* D**56**, 1413–1420. 10.1107/s0907444900010039 11053839

[bb175] Robbins, A. H., McRee, D. E., Williamson, M., Collett, S. A., Xuong, N. H., Furey, W. F., Wang, B.-C. & Stout, C. D. (1991). *J. Mol. Biol.* **221**, 1269–1293. 1942051

[bb176] Robertson, J. H. & Beevers, C. A. (1951). *Acta Cryst.* **4**, 270–275.

[bb177] Robertson, J. M. (1936). *J. Chem. Soc.*, pp. 1195–1209.

[bb178] Robertson, J. M. & Woodward, I. (1940). *J. Chem. Soc.*, pp. 36–48.

[bb179] Rodgers, D. W. (1994). *Structure*, **2**, 1135–1140. 10.1016/s0969-2126(94)00116-2 7704524

[bb180] Rose, J. P., Wang, B.-C. & Weiss, M. S. (2015). *IUCrJ*, **2**, 431–440. 10.1107/S2052252515008337 PMC4491315 26175902

[bb181] Rosenbaum, G., Holmes, K. C. & Witz, J. (1971). *Nature*, **230**, 434–437.

[bb300] Rossmann, M. G. (1960). *Acta Cryst.* **13**, 221–226.

[bb182] Rossmann, M. G. (1961). *Acta Cryst.* **14**, 383–388.

[bb310] Rossmann, M. G. (1990). *Acta Cryst.* A**46**, 73–82. 10.1107/s0108767389009815 2180438

[bb183] Rossmann, M. G. & Blow, D. M. (1961). *Acta Cryst.* **14**, 641–647.

[bb184] Rossmann, M. G. & Blow, D. M. (1962). *Acta Cryst.* **15**, 24–31.

[bb185] Rossmann, M. G. & Blow, D. M. (1963). *Acta Cryst.* **16**, 39–45.

[bb186] Ryu, S.-E., Kwong, P. D., Truneh, A., Porter, T. G., Arthos, J., Rosenberg, M., Dai, X., Xuong, N.-H., Axel, R. & Sweet, R. W. (1990). *Nature*, **348**, 419–426. 10.1038/348419a0 PMC5638305 2247146

[bb187] Saibil, H. R. (2022). *Mol. Cell*, **82**, 274–284. 10.1016/j.molcel.2021.12.016 35063096

[bb189] Sayre, D. (1952). *Acta Cryst.* **5**, 60–65.

[bb188] Sayre, D. (1974). *Acta Cryst.* A**30**, 180–184.

[bb190] Schmid, M. F. & Herriott, J. R. (1976). *J. Mol. Biol.* **103**, 175–190. 10.1016/0022-2836(76)90058-9 957425

[bb191] Schmid, M. F., Herriott, J. R. & Lattman, E. E. (1974). *J. Mol. Biol.* **84**, 97–101. 10.1016/0022-2836(74)90214-9 4830865

[bb207] Schmidt, A., Teeter, M., Weckert, E. & Lamzin, V. S. (2011). *Acta Cryst.* F**67**, 424–428. 10.1107/S1744309110052607 PMC3080141 21505232

[bb192] Schneider, T. R. & Sheldrick, G. M. (2002). *Acta Cryst.* D**58**, 1772–1779. 10.1107/s0907444902011678 12351820

[bb193] Shapiro, L., Doyle, J. P., Hensley, P., Colman, D. R. & Hendrickson, W. A. (1996). *Neuron*, **17**, 435–449. 10.1016/s0896-6273(00)80176-2 8816707

[bb194] Shapiro, L., Fannon, A. M., Kwong, P. D., Thompson, A., Lehmann, M. S., Grübel, G., Legrand, J. F., Als-Nielsen, J., Colman, D. R. & Hendrickson, W. A. (1995). *Nature*, **374**, 327–337. 10.1038/374327a0 7885471

[bb195] Shapiro, L. & Lima, C. D. (1998). *Structure*, **6**, 265–267. 10.1016/s0969-2126(98)00030-6 9551549

[bb196] Sheldrick, G. M., Dauter, Z., Wilson, K. S., Hope, H. & Sieker, L. C. (1993). *Acta Cryst.* D**49**, 18–23. 10.1107/S0907444992007364 15299542

[bb197] Sibanda, B. L., Chirgadze, D. Y., Ascher, D. B. & Blundell, T. L. (2017). *Science*, **355**, 520–524. 10.1126/science.aak9654 28154079

[bb198] Sim, G. A. (1960). *Acta Cryst.* **13**, 511–512.

[bb199] Smith, G. D., Nagar, B., Rini, J. M., Hauptman, H. A. & Blessing, R. H. (1998). *Acta Cryst.* D**54**, 799–804. 10.1107/s0907444997018805 9757093

[bb200] Smith, J. L., Hendrickson, W. A. & Addison, A. W. (1983). *Nature*, **303**, 86–88. 10.1038/303086a0 6843663

[bb201] Smith, J. L., Hendrickson, W. A., Honzatko, R. B. & Sheriff, S. (1986). *Biochemistry*, **25**, 5018–5027. 10.1021/bi00366a008 3768328

[bb202] Spatzal, T., Schlesier, J., Burger, E. M., Sippel, D., Zhang, L., Andrade, S. L., Rees, D. C. & Einsle, O. (2016). *Nat. Commun.* **7**, 10902. 10.1038/ncomms10902 PMC4793075 26973151

[bb203] Srinivasan, R. (1966). *Acta Cryst.* **20**, 143–144.

[bb204] Srinivasan, R. & Ramachandran, G. N. (1965). *Acta Cryst.* **19**, 1008–1014.

[bb205] Stauber, D. J., DiGabriele, A. D. & Hendrickson, W. A. (2000). *Proc. Natl Acad. Sci. USA*, **97**, 49–54. 10.1073/pnas.97.1.49 PMC26614 10618369

[bb206] Staudenmann, J.-L., Hendrickson, W. A. & Abramowitz, R. (1989). *Rev. Sci. Instrum.* **60**, 1939–1942.

[bb208] Steitz, T. A. (1968). *Acta Cryst.* B**24**, 504–507.

[bb209] Sussman, J. L. (1985). *Methods Enzymol.* **115**, 271–303. 10.1016/0076-6879(85)15022-6 2417093

[bb210] Teeter, M. M. & Hendrickson, W. A. (1979). *J. Mol. Biol.* **127**, 219–223. 10.1016/0022-2836(79)90242-0 430565

[bb211] Templeton, D. H. & Templeton, L. K. (1982). *Acta Cryst.* A**38**, 62–67.

[bb212] Templeton, D. H., Templeton, L. K., Phillips, J. C. & Hodgson, K. O. (1980). *Acta Cryst.* A**36**, 436–442.

[bb213] Templeton, L. K. & Templeton, D. H. (1988). *Acta Cryst.* A**44**, 1045–1051. 10.1107/s0108767388007810 3271115

[bb214] Terwilliger, T. C. (2000). *Acta Cryst.* D**56**, 965–972. 10.1107/S0907444900005072 PMC2792768 10944333

[bb215] Terwilliger, T. C., Afonine, P. V., Liebschner, D., Croll, T. I., McCoy, A. J., Oeffner, R. D., Williams, C. J., Poon, B. K., Richardson, J. S., Read, R. J. & Adams, P. D. (2023). *Acta Cryst.* D**79**, 234–244. 10.1107/S205979832300102X PMC9986801 36876433

[bb216] Thomson, J. J. (1906).* Conduction of Electricity through Gases*, 2nd ed. Cambridge University Press.

[bb217] Tollin, P. (1969). *J. Mol. Biol.* **45**, 481–490. 10.1016/0022-2836(69)90307-6 5386655

[bb218] Turner, M. A., Yuan, C. S., Borchardt, R. T., Hershfield, M. S., Smith, G. D. & Howell, P. L. (1998). *Nat. Struct. Biol.* **5**, 369–376. 10.1038/nsb0598-369 9586999

[bb219] Vand, V. & Pepinsky, R. (1957). *Acta Cryst.* **10**, 563–567.

[bb220] Vecchioni, S., Lu, B., Livernois, W., Ohayon, Y. P., Yoder, J. B., Yang, C., Woloszyn, K., Bernfeld, W., Anantram, M. P., Canary, J. W., Hendrickson, W. A., Rothschild, L. J., Mao, C., Wind, S. J., Seeman, N. C. & Sha, R. (2023). *Adv. Mater.* **35**, 2210938. 10.1002/adma.202210938 37268326

[bb221] Venkatesan, K., Dale, D., Hodgkin, D. C., Nockolds, C. E., Moore, F. H. & O’Connor, B. H. (1971). *Proc. R. Soc. London A*, **323**, 455–487.

[bb222] Wagner, A., Duman, R., Henderson, K. & Mykhaylyk, V. (2016). *Acta Cryst.* D**72**, 430–439. 10.1107/S2059798316001078 PMC4784674 26960130

[bb223] Wang, B.-C. (1985). *Methods Enzymol.* **115**, 90–112. 10.1016/0076-6879(85)15009-3 4079800

[bb224] Ward, K. B., Hendrickson, W. A. & Klippenstein, G. L. (1975). *Nature*, **257**, 818–821. 10.1038/257818a0 1186872

[bb225] Warkentin, M. & Thorne, R. E. (2010). *Acta Cryst.* D**66**, 1092–1100. 10.1107/S0907444910035523 PMC2954455 20944242

[bb226] Warren, B. & Bragg, W. L. (1929). *Z. Kristallogr.* **69**, 168–193.

[bb227] Watson, J. D. (1972). *Structure and Function of Proteins at the Three-Dimensional Level.* New York: Cold Spring Harbor Laboratory Press.

[bb228] Watson, J. D. & Crick, F. H. C. (1953). *Nature*, **171**, 737–738. 10.1038/171737a0 13054692

[bb229] Weeks, C. M., Hauptman, H. A., Smith, G. D., Blessing, R. H., Teeter, M. M. & Miller, R. (1995). *Acta Cryst.* D**51**, 33–38. 10.1107/S090744499400925X 15299333

[bb230] Weinert, T., Olieric, V., Waltersperger, S., Panepucci, E., Chen, L., Zhang, H., Zhou, D., Rose, J., Ebihara, A., Kuramitsu, S., Li, D., Howe, N., Schnapp, G., Pautsch, A., Bargsten, K., Prota, A. E., Surana, P., Kottur, J., Nair, D. T., Basilico, F., Cecatiello, V., Pasqualato, S., Boland, A., Weichenrieder, O., Wang, B.-C., Steinmetz, M. O., Caffrey, M. & Wang, M. (2015). *Nat. Methods*, **12**, 131–133. 10.1038/nmeth.3211 25506719

[bb231] Weis, W. I., Kahn, R., Fourme, R., Drickamer, K. & Hendrickson, W. A. (1991). *Science*, **254**, 1608–1615. 10.1126/science.1721241 1721241

[bb232] Weiss, M. S., Sicker, T., Djinovic-Carugo, K. & Hilgenfeld, R. (2001). *Acta Cryst.* D**57**, 689–695. 10.1107/s0907444901003572 11320309

[bb234] Williams, J. C., Sue, C., Banting, G. S., Yang, H., Glerum, D. M., Hendrickson, W. A. & Schon, E. A. (2005). *J. Biol. Chem.* **280**, 15202–15211. 10.1074/jbc.M410705200 15659396

[bb235] Williams, J. C., Xie, H. & Hendrickson, W. A. (2005). *J. Biol. Chem.* **280**, 21981–21986. 10.1074/jbc.M414643200 15701632

[bb233] Williams, J. C., Roulhac, P. L., Roy, A. G., Vallee, R. B., Fitzgerald, M. C. & Hendrickson, W. A. (2007). *Proc. Natl Acad. Sci. USA*, **104**, 10028–10033. 10.1073/pnas.0703614104 PMC1885999 17551010

[bb236] Wilson, I. A., Skehel, J. J. & Wiley, D. C. (1981). *Nature*, **289**, 366–373. 10.1038/289366a0 7464906

[bb882] Winkler, F. K., Schutt, C. E., Harrison, S. C. & Bricogne, G. (1977). *Nature*, **265**, 509–513. 10.1038/265509a0 834302

[bb237] Wu, H., Lustbader, J. W., Liu, Y., Canfield, R. E. & Hendrickson, W. A. (1994). *Structure*, **2**, 545–558. 10.1016/s0969-2126(00)00054-x 7922031

[bb238] Xiao, R., Anderson, S., Aramini, J., Belote, R., Buchwald, W. A., Ciccosanti, C., Conover, K., Everett, J. K., Hamilton, K., Huang, Y. J., Janjua, H., Jiang, M., Kornhaber, G. J., Lee, D. Y., Locke, J. Y., Ma, L., Maglaqui, M., Mao, L., Mitra, S., Patel, D., Rossi, P., Sahdev, S., Sharma, S., Shastry, R., Swapna, G. V. T., Tong, S. N., Wang, D., Wang, H., Zhao, L., Montelione, G. T. & Acton, T. B. (2010). *J. Struct. Biol.* **172**, 21–33. 10.1016/j.jsb.2010.07.011 PMC4110633 20688167

[bb239] Yamaguchi, H. & Hendrickson, W. A. (1996). *Nature*, **384**, 484–489. 10.1038/384484a0 8945479

[bb240] Yang, C., Pflugrath, J. W., Courville, D. A., Stence, C. N. & Ferrara, J. D. (2003). *Acta Cryst.* D**59**, 1943–1957. 10.1107/s0907444903018547 14573949

[bb241] Yang, T., Liu, Q., Kloss, B., Bruni, R., Kalathur, R. C., Guo, Y., Kloppmann, E., Rost, B., Colecraft, H. M. & Hendrickson, W. A. (2014). *Science*, **346**, 355–359. 10.1126/science.1259723 PMC4341822 25324390

[bb242] Yang, W., Hendrickson, W. A., Crouch, R. J. & Satow, Y. (1990). *Science*, **249**, 1398–1405. 10.1126/science.2169648 2169648

[bb243] Zeng, B., Mou, T. C., Doukov, T. I., Steiner, A., Yu, W., Papasergi-Scott, M., Tall, G. G., Hagn, F. & Sprang, S. R. (2019). *Structure*, **27**, 1137–1147. 10.1016/j.str.2019.04.013 PMC6610710 31155309

[bb244] Zhang, K. Y. J. & Main, P. (1990). *Acta Cryst.* A**46**, 41–46.

[bb245] Zhang, Z. & Hendrickson, W. A. (2010). *J. Mol. Biol.* **400**, 335–353. 10.1016/j.jmb.2010.04.049 PMC3461309 20435045

[bb246] Zhang, Z., Liu, Q. & Hendrickson, W. A. (2014). *FEBS J.* **281**, 4263–4279. 10.1111/febs.12904 PMC4169315 24995510

[bb247] Zhu, X., Zhao, X., Burkholder, W. F., Gragerov, A., Ogata, C. M., Gottesman, M. E. & Hendrickson, W. A. (1996). *Science*, **272**, 1606–1614. 10.1126/science.272.5268.1606 PMC5629921 8658133

